# 4D Multimodal Nanomedicines Made of Nonequilibrium Au–Fe Alloy Nanoparticles

**DOI:** 10.1021/acsnano.0c03614

**Published:** 2020-09-02

**Authors:** Veronica Torresan, Daniel Forrer, Andrea Guadagnini, Denis Badocco, Paolo Pastore, Maurizio Casarin, Annabella Selloni, Diego Coral, Marcelo Ceolin, Marcela B. Fernández van Raap, Alice Busato, Pasquina Marzola, Antonello E. Spinelli, Vincenzo Amendola

**Affiliations:** † Department of Chemical Sciences, University of Padova, Padova, I-35131 Italy; ‡ CNR−ICMATE, Padova, I-35131 Italy; § Department of Chemistry, Princeton University, Princeton, New Jersey 08544, United States; ∥ Departamento de Fisica, Universidad del Cauca, 193577 Popayán, Colombia; ⊥ Departamento de Quımica, Facultad de Ciencias Exactas, Universidad Nacional de La Plata−CONICET, Instituto de Investigaciones Fisicoquımicas Teoricas y Aplicadas (INIFTA), La Plata, 1900 Argentina; # Departamento de Física Facultad de Ciencias Exactas, Universidad Nacional de La Plata−CONICET, Instituto de Física La Plata (IFLP), La Plata, 1900 Argentina; △ Department of Computer Science, University of Verona, Verona, 37134 Italy; ▽ Experimental Imaging Centre, IRCCS San Raffaele Scientific Institute, Milan, 20132 Italy

**Keywords:** Au nanoparticles, Fe nanoparticles, alloys, nanomedicine, degradable materials, CT, MRI

## Abstract

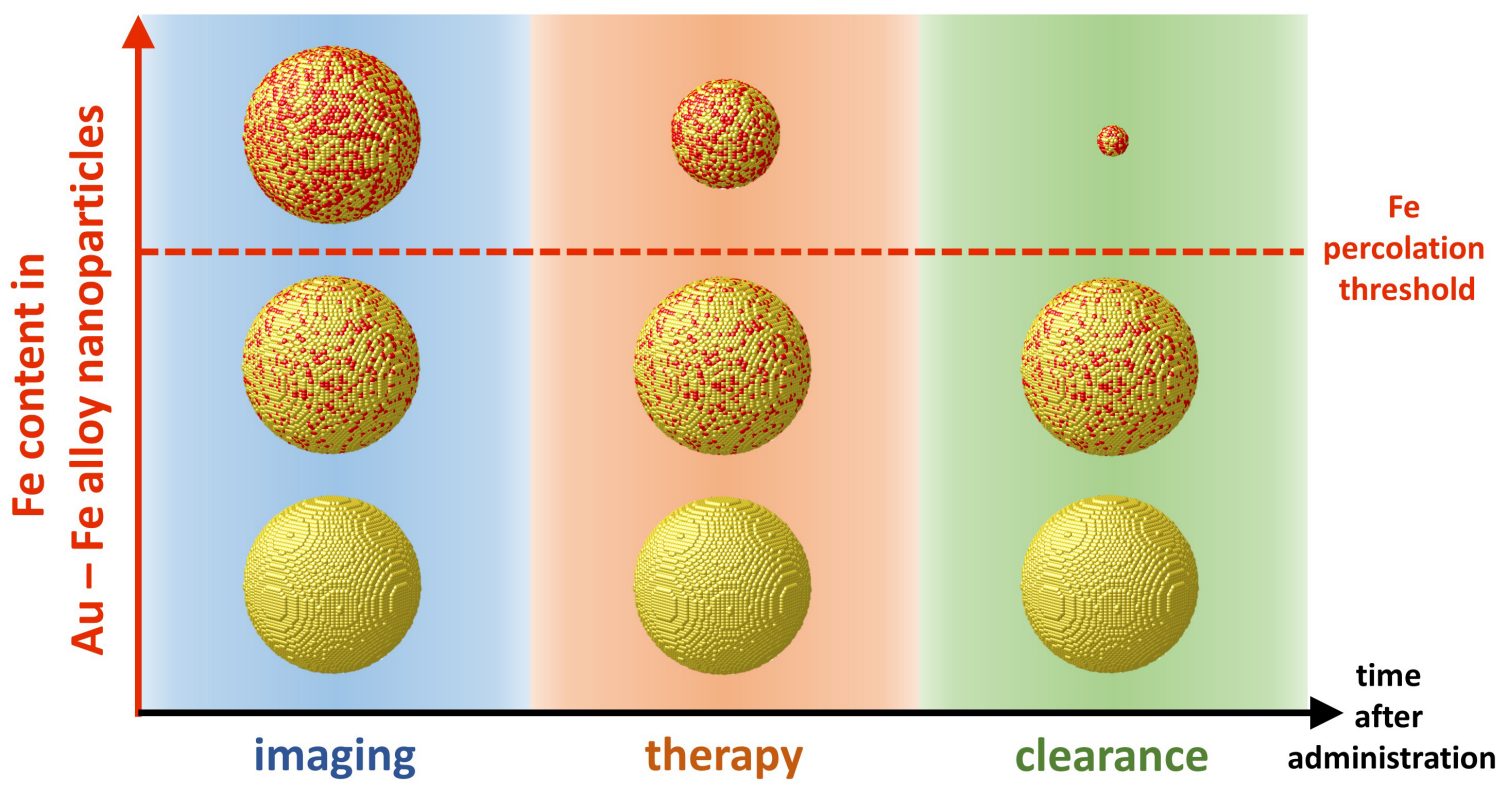

Several examples of nanosized therapeutic and imaging agents have been proposed to date, yet for most of them there is a low chance of clinical translation due to long-term *in vivo* retention and toxicity risks. The realization of nanoagents that can be removed from the body after use remains thus a great challenge. Here, we demonstrate that nonequilibrium gold–iron alloys behave as shape-morphing nanocrystals with the properties of self-degradable multifunctional nanomedicines. DFT calculations combined with mixing enthalpy-weighted alloying simulations predict that Au–Fe solid solutions can exhibit self-degradation in an aqueous environment if the Fe content exceeds a threshold that depends upon element topology in the nanocrystals. Exploiting a laser-assisted synthesis route, we experimentally confirm that nonequilibrium Au–Fe nanoalloys have a 4D behavior, that is, the ability to change shape, size, and structure over time, becoming ultrasmall Au-rich nanocrystals. *In vivo* tests show the potential of these transformable Au–Fe nanoalloys as efficient multimodal contrast agents for magnetic resonance imaging and computed X-ray absorption tomography and further demonstrate their self-degradation over time, with a significant reduction of long-term accumulation in the body, when compared to benchmark gold or iron oxide contrast agents. Hence, Au–Fe alloy nanoparticles exhibiting 4D behavior can respond to the need for safe and degradable inorganic multifunctional nanomedicines required in clinical translation.

The rapid progress of nanomedicine has led to a multitude of sophisticated inorganic and hybrid nanomaterials with excellent diagnostic and therapeutic performances, which hold promise to revolutionize medical treatments in the near future.^1−3^ However, the long-term biopersistence caused by limited or null degradability, with the resulting accumulation in the body, is a critical issue that makes the clinical translation of inorganic nanomedicines, namely, of nanosized inorganic therapeutic and imaging agents, very rare.^4−6^ Gold- and iron-based nanostructures are one such widely exploited and highly attractive class of inorganic nanomedicines.^7−10^ Au nanoparticles (NPs) are well known for being nondegradable^11,12^ and for their ability to withstand the corrosive intracellular environment.^13^ Their biopersistence *in vivo* with substantial retention in the liver and spleen has been verified even after 10 months,^7^ although slow dissolution dynamics in specific lysosomal environments has been reported.^14^

Several investigations have assessed the biocompatibility and long-term biopersistence of nanostructures based on iron oxides, demonstrating how these inorganic materials are inherently biodegradable and are metabolized in the organism through the mediation of transferrin.^3,15−17^ However, clinically approved iron oxide contrast agents (CAs) are known to be retained *in vivo* in a quantity comparable to the administered dose even after 70 days.^18^ This occurs because their surface is capped with a polymeric shell that hampers inorganic core dissolution.^11,19,20^

Even in the case of elements that are generally considered safe and biocompatible such as Au and Fe, long-term biopersistence is an issue because it may kindle chronic inflammatory reactions, elicit oxidative stress,^7^ or impair the phagocytic activity of the mononuclear phagocyte system (MPS).^2,20,21^ Therefore, ideal nanomedicines should degrade into nontoxic fragments that are easily cleared by the body after their function at specific sites is terminated.^2,3^ Besides, a nanoagent for cancer treatment should have a size in the 50–200 nm range to achieve prolonged blood circulation time without immediate renal clearance and high tumor accumulation by the enhanced permeation and retention effect.^22^ However, they should also be smaller than 20 nm to penetrate and homogeneously distribute inside the target tissue.^22−24^

In the present study, we seek to address these problems by developing a nanomedicine agent featuring the multimodal distinctive properties of gold–iron bimetallic NPs together with 4D behavior, that is the ability to change shape, size, and structure over time, in a physiological environment.^25,26^ In this case, the Au–Fe bimetallic NPs exhibit degradability and size reduction into Au-rich nanocrystals smaller than 10 nm. In contrast to previous studies on degradable inorganic nanostructures,^3,4,21,27−29^ here only biocompatible Au and Fe elements are used. Bimetallic Au–Fe nanostructures have been often investigated as a promising class of multifunctional biocompatible inorganic materials for diagnostic and therapeutic applications.^8,30^ In fact, the 4D Au–Fe nanomedicines retain the multifunctionality and versatility of monometallic NPs, such as the easy surface chemistry of Au and its high X-ray attenuation cross-section useful for computed X-ray tomography (CT), combined with the high magnetic moment of Fe, exploitable for magnetic resonance imaging (MRI), and its degradability in physiological environment. To show that nonequilibrium Au–Fe solid solutions can be 4D nanomedicine agents, we started from a theoretical analysis based on density functional theory (DFT) calculations of iron reactivity and atomic diffusivity in the alloy, combined with topological predictions of atomic structures based on mixing enthalpy-weighted alloy formation. Next, a laser-assisted synthetic route allowed us to bypass the thermodynamic limitations and obtain nonequilibrium Au–Fe nanoalloys in a range of compositions from pure Au up to the threshold for experimental observation of quantitative nanomedicine self-degradation in a physiological environment. The inherent multifunctionality of these Au–Fe nanoalloys was exploited to further demonstrate *in vivo* their potential as an effective multimodal CA in CT and MRI, which are the two most common total-body clinical imaging techniques and have complementary spatial resolution and sensitivity with soft and dense tissues.^31,32^

## Results and Discussion

### Numerical Simulations of Degradable Nanoalloy Structures

**Figure 1:**
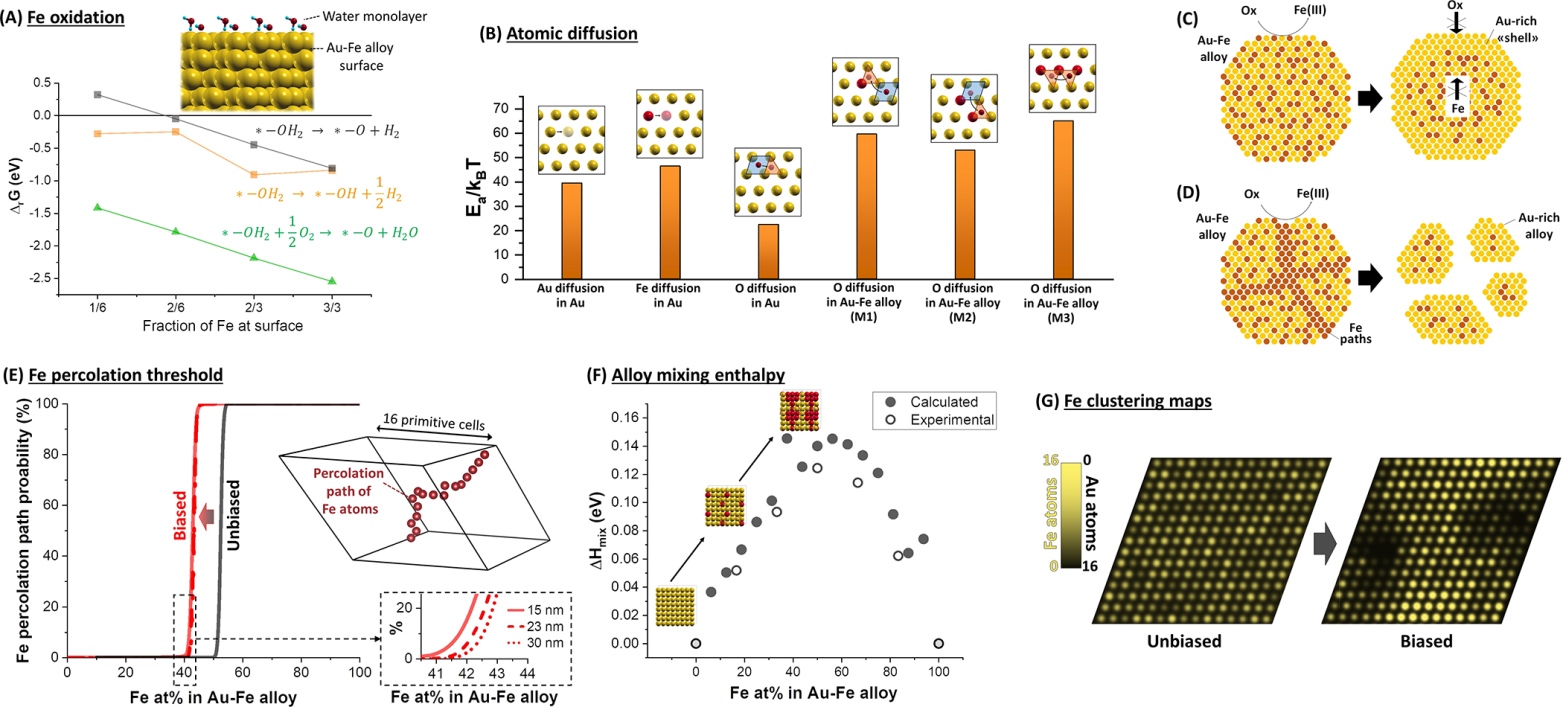
DFT calculations and numerical simulations. (A) Gibbs free energies of the iron oxidation reactions reported as a function of the Fe amount at the surface; * indicates an Fe surface site. (B) Energy barriers for the diffusion of Au, Fe, and O atomic species in alloy bulk, computed using the climbing-image nudged elastic band (NEB) method (see SI) and different alloy models. (C) Pictorial model of the nanoalloy evolution in water media for an alloy composition below the percolation threshold, for which the oxidation of surface Fe leads to passivation and (D) for an alloy composition above the percolation threshold, for which oxidation can proceed along percolation paths. (E) Percolation threshold as a function of the alloy composition for τ = ∞ (black line) or 0.26 eV (red line); inset represents an example of a percolation path. The threshold has been evaluated also as a function of slab size, evidencing that it is influent for only a few at. % on the result. This is appreciable from the zoom-in black dashed inset (continuous red line: 15 nm slab; red dashed lines: 23 nm slab; red dotted line: 30 nm slab). (F) Mixing enthalpy of the alloy. (G) Distribution of Fe atoms in a supercell, obtained by counting Fe atoms along a close-packed direction, in the perfectly random alloy (unbiased) and in an alloy model where segregation is allowed (biased).

While one of the prominent advantages of iron oxide-based NPs is biodegradability,^19,20^ Au–Fe alloys with an iron content up to 13 at. % are indefinitely stable in water and resist heat treatment with strong iron-chelating agents such as EDTA.^8,33^ Nonetheless, computed free energies of iron oxidation at the surface of Au–Fe alloys (Figure 1A) show that, similarly to what is found in pure metallic iron, Fe readily reacts with adsorbed water molecules or dissolved atmospheric oxygen to form oxidized iron species that can be easily removed in a physiological environment.^16^ The change in Gibbs free energy clearly indicates that the oxidation of Fe by water with formation of surface hydroxides is favored at any alloy composition, whereas the formation of surface oxide is possible only when the amount of Fe exceeds a certain threshold. Conversely, oxide formation through reaction with molecular oxygen is always favored. According to these results, surface passivation must be responsible for the stability of iron-poor Au–Fe alloys observed in previous studies.^8,33^ The interior of a material is passivated against corrosion only when its surface inhibits the diffusion of ions and molecules involved in the chemical reactions of dissolution.^30,34−36^ In fact, the estimated energy barriers for the diffusion of Au, Fe, and O atoms in the AuFe face centered cubic (fcc) lattice (Figure 1B) confirmed the hypothesis of surface passivation in Au–Fe alloys. For all the mechanisms considered (see Figure 1B and Section S3 in the Supporting Information, SI), energy barriers are too high, on the order of tens of *k*_B_*T* for both O and Fe atoms, to allow atomic mobility at room temperature.

From an atomistic point of view, corrosion of the Au–Fe alloy can occur through different mechanisms: (i) propagation of the oxidation by means of oxygen migration through the alloy lattice, (ii) Fe migration toward the surface to sustain superficial oxidation, or (iii) pitting of the surface and consequent exposure of surface Fe to the aqueous environment. However, if atomic diffusion is frozen in the Au fcc cell (Figure 1B,C), the only possibility for Fe atoms to be oxidized and released into the surrounding liquid is that they are part of a percolative path of pure Fe embedded in the compact layer of the alloy (Figure 1D),^34,35,37^ where etching can proceed by pitting, as found for pure iron nanostructures. In thermodynamically stable bimetallic alloys such as Au–Ag, where the two metals are randomly distributed in the crystalline lattice sites, the formation of “percolation paths” depends only on the composition.^34,35,38^ For instance, when a topological model to identify the parting limit for dealloying (see Section S5 in SI) is applied to random substitutional Au–Fe alloys, the iron percolation paths between two sides of the simulation cell appear in 100% of the considered structures only when the Fe content exceeds 54 at. % (Figure 1E). In these calculations, a “percolative” Fe path is formed when all its iron atoms have at least 8 next neighbors of the same type, over a total of 12 for an fcc lattice structure, in agreement with previous studies on dealloying in thermodynamically stable solid solutions.^35^ Noteworthy, the trend has a well-known^35^ steep dependence on alloy composition, such that no percolation is predicted already below 52 at. % of Fe. However, as clearly evidenced by the plot of mixing enthalpy *versus* composition (Figure 1F), the Au–Fe alloy is a nonequilibrium system with a thermodynamic tendency to element segregation and phase separation into pure Au and Fe domains,^39^ which is inhibited at room temperature only because of the high diffusion barriers inside the metal lattice.^40,41^ When this tendency to homoatomic clustering is included in the topological model, by weighting the lattice site occupation with a function of the mixing enthalpy and using a thermal disorder parameter τ of 0.26 eV, the threshold for the appearance of percolative paths in the alloy is found at a lower iron fraction of 43% (red line in Figure 1E). Direct evidence of iron segregation is provided by the plot of Au and Fe atom probability along a specific direction, as shown in Figure 1G. A series of calculations of the threshold for 100% of iron percolation paths *versus* the size of the slab evidenced a variation of only a few at. % in the 15–30 nm size range for any value of τ (Figure 1E and Figure S4 in the SI). This suggests that the phenomenon should be independent from the size and polydispersity of NPs in the range of few tens of nm.

### Experimental Assessment of 4D Behavior in Au–Fe Alloy NPs

Based on the above theoretical analysis, the percolative faults of cleavable iron inside Au–Fe crystals can be achieved by adopting out-of-equilibrium synthetic conditions while, at the same time, allowing some atomic diffusivity at the sub-nanometric range. These crystals should behave like transformable bimetallic objects that spontaneously evolve into smaller Au-rich nanocrystals releasing Fe ions to the environment. To provide experimental verification for this hypothesis, we applied a synthetic strategy based on laser ablation in liquid (LAL) to obtain nonequilibrium Au–Fe nanoalloys in the desired range of compositions.^33,42,43^ LAL consists in the ablation of a bulk plate immersed in a liquid solution, by using focused pulsed laser beams, and it is renowned as a versatile and green route to achieve colloids of NPs in one step, without the need for chemical precursors and avoiding undesired or toxic byproducts.^33,42,43^ This is important for bioapplications, since the colloids are pure and the particle surface is available for direct conjugation with the desired biomolecules.^33,42^ Besides, LAL is a cost-effective approach because it only requires raw materials and pure liquids, and it relies on a self-standing setup that is amenable to scale-up in a remotely controllable continuous flow-synthesis configuration.^44^

Taking advantage of the versatility of our synthetic protocol, we prepared a set of NP samples with composition ranging from pure Au to Au(79)Fe(21), Au(70)Fe(30), and Au(50)Fe(50), as assessed by inductively coupled plasma-assisted mass spectrometry (ICP-MS, see Table S7 in SI). To shift the alloy composition in the nonequilibrium region of the Au–Fe phase diagram, LAL was performed with bulk metal targets of Au alloyed with increasing amounts of iron, while keeping unchanged all the other synthetic parameters. So obtained Au and Au–Fe alloy NPs all share the same surface coating of biocompatible polyethylene glycol (PEG). In fact, thiolated PEG was dissolved in the liquid used for LAL, so that the coating with PEG happens simultaneously to laser synthesis of NPs, by the spontaneous formation of S–Au chemical bonds. PEG coating is renowned for conferring colloidal stability in physiological environment and for limiting adsorption of serum proteins such as albumin or opsonins.^45−47^ This is a requisite to achieve appreciable biodistribution and biopersistence and to limit sequestration by the MPS.^3,46,48^

**Figure 2:**
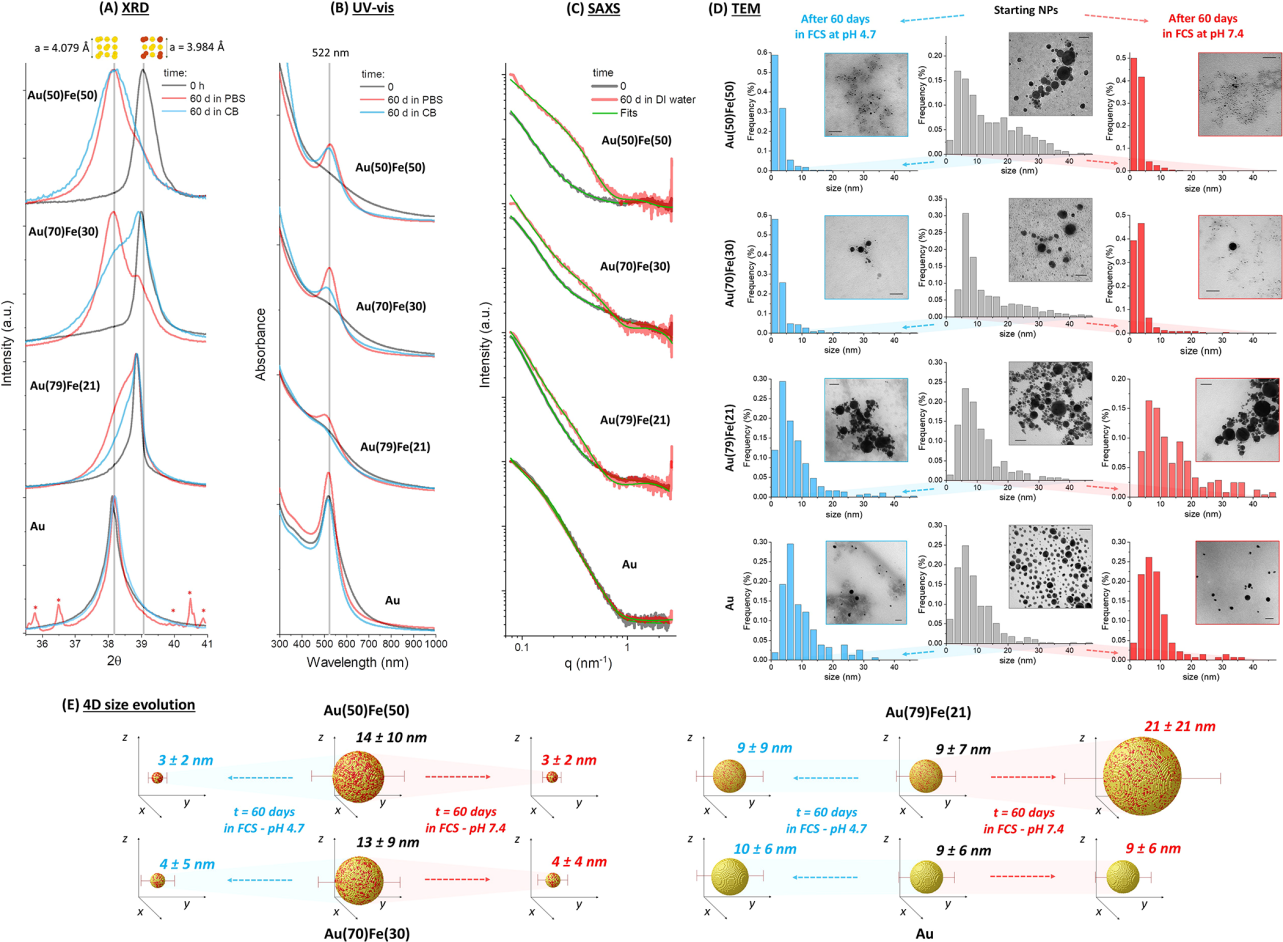
4D structural and size evolution. XRD (A), UV–vis (B), SAXS (C), and TEM (D) analysis on Au and AuFe samples before and after aging for 60 days in different environments: for XRD and UV–vis in PBS or citrate buffer (CB), for SAXS in distilled water, for TEM in FCS at pH 7.4 and 4.7. In all cases, the pure Au sample shows negligible modifications, while the Au–Fe alloy sample exhibits increasing structural evolution for increasing Fe content. In (A), the XRD peaks due to residual buffer salts in the Au NPs sample incubated 60 days in PBS are denoted with red asterisks. In (D), the scale bar of TEM images is 50 nm. (E) Sketch resuming the evolution of average NP size described in (D) after 60 days, where the 4D transformable nature of the Au(50)Fe(50) and Au(30)Fe(70) samples is well evidenced. In fact, while stable NPs are defined by their 3D shape, the Au–Fe alloy NPs transform over time, requiring a fourth dimension (time) to be properly identified.

X-ray diffraction (XRD) analysis confirmed the formation of Au–Fe alloys (see Figure 2A). The introduction of Fe in the nanoalloys is associated with two main structural effects, namely, (i) the reduction of the cell parameter, ascribable to the lower atomic radius of iron compared to gold and its distribution as a random substitutional dopant in the fcc lattice of Au, and (ii) the increase of peak width, which is correlated to the size reduction of monocrystalline domains. The average crystallite size, evaluated by the Scherrer equation, decreases from the 14–18 nm of Au, Au(79)Fe(21), and Au(70)Fe(30) samples to 9 nm in the Au(50)Fe(50) sample (Table S8). Considering the increasing mixing enthalpy of the Au–Fe system, the observation of smaller crystalline domains in the Au(50)Fe(50) sample suggests a certain level of crystalline disorder ascribable to the thermodynamic tendency to iron segregation at grain borders, in agreement with the topological model reported in Figure 1G. To obtain further evidence on the crystalline disorder in the Au(50)Fe(50) sample, we performed high-resolution transmission electron microscopy (HRTEM), scanning-TEM (STEM), high angle annular dark field (HAADF), and bidimensional energy dispersive spectroscopy (EDS) analysis on a set of individual NPs (Figures S6 and S7 in SI). HRTEM images (Figure S6 in SI) confirmed that the nanoalloys have polycrystalline structure, with a large number of grain borders and defects. An inhomogeneous electronic contrast (*i*.*e*., darker and clearer regions) is also appreciable inside the single nanoparticle. The contrast inhomogeneity is typically encountered in bimetallic NPs featuring a nonhomogeneous distribution of elements with different atomic number. This is confirmed by the images acquired with the STEM-HAADF dark field (DF) modality (Figure S7 in SI), which is renowned to provide a contrast map that depends on atom type and distribution, while being much less influenced than HRTEM by the orientation of the crystalline domains inside NPs. Despite this inhomogeneity, the bidimensional STEM-EDS images of the Au M-line and the Fe K-line confirmed the colocalization of the two elements inside each single NP, which was already unequivocally evidenced by the XRD analysis.

Given these favorable structural features for the 4D behavior, our first goal was to monitor the evolution of the alloys in the typical physiological environment (PBS at pH 7.4) and in conditions mimicking the interior of lysosomes (citrate buffer at pH 4.7), with a procedure inspired by previous investigations on nanostructured iron oxides and noble metals.^11,15,30^ To this purpose, the nanoalloys were incubated at 37 °C in the basic and acidic buffers for 60 days, after which the XRD spectra were collected again (Figure 2A). From the shift in the peaks originated from the (111) planes of the fcc cell (at 2θ ≈ 38–39°), it is evident how the alloys with the highest content of Fe underwent significant structural modifications, with cell parameters after 60 days that are close to those of pure Au (see Table S8 in SI).^42^ Therefore, dealloying was possible well below 54 at. % of Fe, as predicted by the topological model for iron percolation paths in nonequilibrium alloys. On the other hand, the peak positions in the XRD pattern of Au remained unchanged, and those in the pattern of the Au(79)Fe(21) NPs underwent only partial modification with the appearance of a second, less intense, component with intermediate cell parameter between pure Au and pristine Au(79)Fe(21) NPs.

To extract additional insights about composition dynamics, the optical properties of Au–Fe alloys were monitored (see Figure 2B). Au NPs possess a distinctive absorption peak in the visible range (*ca*. 520 nm) due to the excitation of the localized surface plasmon resonance (LSPR) of the metal.^49^ However, the position and intensity of this absorption peak depend strongly on Fe doping in the Au lattice, because of deep changes in the electronic band structure of the metal.^50,51^ This is appreciable from the progressive dumping of the LSPR band when going from pure Au to Au(50)Fe(50) NPs dispersed in water (Figure 2B). When the absorption spectra are collected after aging for two months at 37 °C in basic and acidic aqueous environments (same as XRD analysis), the composition-dependent dealloying is clear; since no dramatic changes are observed in pure Au NPs, minimal spectral modifications are found in the Au(79)Fe(21) NPs, while sharp plasmon bands appeared in the Au(70)Fe(30) and Au(50)Fe(50) samples. To assess if the compositional transformation of the nanoalloys is accompanied by a size evolution, as desirable for nanomedicine applications, we performed small-angle X-ray scattering (SAXS) experiments (Figure 2C). In this case, the four samples were aged for two months in pure water at 37 °C instead of a physiological environment at a given pH, to avoid the high ionic strength of the buffers, which may induce extensive particle agglomeration and possible interference with the SAXS analysis. As appreciable from Figure 2C, the change of SAXS profiles over time is strongly correlated with the composition of the alloy. In particular, the size evolution extracted from the fitting of the SAXS curves (Figure S8 in SI) is large for the Au(50)Fe(50) NPs and not appreciable for the pure Au NPs, with the Au(70)Fe(30) samples exhibiting intermediate changes between the two extreme cases, and the Au(79)Fe(21) sample, which underwent particle growth in the same period.

To further confirm this finding, the size evolution of the four samples was measured directly by transmission electron microscopy (TEM) after aging in biological fluids such as fetal calf serum (FCS) at physiological (7.4) and lysosomal (4.7) pH (Figure 2D). Biological fluids like serum are multicomponent environments,^12,15,16,19^ rich in iron-complexing chemical species that can accelerate the dissolution of the nanoalloys. These fluids also contain proteins that may adsorb on particle surfaces by influencing the size evolution over time in two opposite ways, *i*.*e*., by hampering dealloying or by avoiding coalescence of the Au-rich fragments into larger agglomerates. In this case, the average size and relative standard deviation of the Au(50)Fe(50) sample, extracted from the TEM measured size distributions, undergo a big reduction over time, from 14 ± 10 nm to 3 ± 2 nm, in both pH conditions (see Figure 2E). The extent of size and standard deviation reduction in the four samples is correlated with the iron content, confirming the trend of SAXS experiments. In more detail, pure Au NPs maintained the initial size of 9 ± 6 nm after 2 months (10 ± 6 nm at pH 4.7 and 9 ± 6 nm at pH 7.4), while Au(79)Fe(21) NPs grew at basic pH (from 9 ± 7 nm to 21 ± 21 nm) and remained unchanged at acidic pH (9 ± 9 nm). The Au(70)Fe(30) sample exhibited size reduction from 13 ± 9 nm to 4 ± 4 nm (pH 7.4) and 4 ± 5 nm (pH 4.7), although a fraction of large NPs was still present after 60 days, which is apparent from the tail in the histograms of Figure 2D. In all samples, the initial size range of the NPs is such that the experiments could provide confirmation to the theoretical predictions about the size-independent threshold for 100% of iron percolation paths for any value of τ (see Figure S4 in SI).

**Figure 3:**
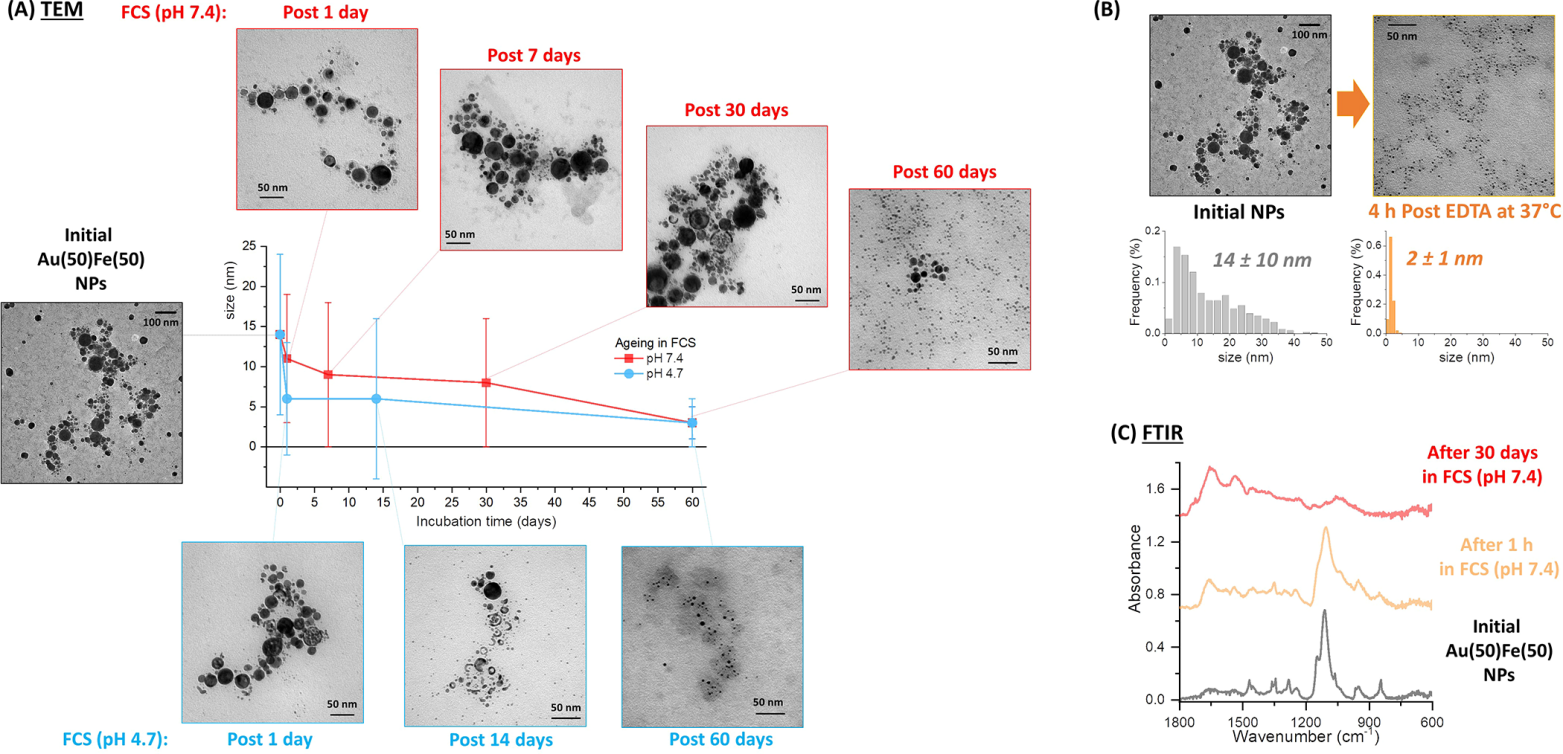
4D size evolution in different environments. (A) TEM analysis at different time points for the Au(50)Fe(50) sample in FCS at pH 7.4 and 4.7. (B) Size distribution and representative TEM image of Au(50)Fe(50) NPs before and after 4 h incubation at 37 °C with EDTA in 20% v/v FCS/water. (C) FTIR of the Au(50)Fe(50) sample before and after aging in FCS (pH 7.4) for 1 h and 30 days. The vibrations of the PEG coating dominate the spectra before and after 1 h, but disappear in the spectrum after 30 days, where only vibrations ascribable to serum proteins are found.

As summarized in Figure 2E, the stable Au NPs are defined by their 3D shape at any time, while the Au–Fe alloy NPs transform, requiring a fourth dimension (time) to be properly identified. To gather more information on the 4D behavior of the Au(50)Fe(50) NPs, which are the most promising for nanomedicine applications, this sample was monitored by TEM at different time points after incubation in FCS solution at physiologic (7.4) and lysosomal (4.7) pH (Figure 3A and Figure S9 for size histograms at each time point). The results show a more rapid size evolution in lysosome-like conditions than at physiological pH, although the average size after 2 months is equivalent. Noticeably, hollow NPs are found in the initial stages of alloy degradation, and nanocrescents are frequently found at longer times, before reaching the final ultrasmall NP morphology. The appearance of hollow structures is typical of oxidative etching and dissolution of a less noble metal in an alloy.^13,52,53^ The amount of dissolved Fe and Au metals was also quantified by ICP-MS at different time points (Figure S10 in SI), confirming the faster degradation of NPs at lysosomal pH and indicating that Fe is dissolved in the FCS solution rich in iron-complexing compounds, while a negligible fraction of Au is dispersed in complexes smaller than the dialysis threshold of 3 kDa.

Of great relevance for clinical applications, the 4D behavior of Au(50)Fe(50) NPs can be triggered by an exogenous chemical stimulus, such as the addition of disodium ethylenediaminetetraacetic acid (EDTA), which is a biocompatible compound with high chemical affinity for metal ions. For instance, the addition of EDTA at the same concentration of typical clinical detoxification procedures for metals^54^ resulted in size reduction of the Au(50)Fe(50) NPs down to 2 ± 1 nm in only 4 h at 37 °C (Figure 3B). Also, this is another confirmation that cleavage of iron from the nanoparticles is facilitated by iron-complexing species, as indicated in the literature.^15,16,20^

The size and structure evolution of the Au(50)Fe(50) NPs must imply some kind of transformation also at the particle surface, which is initially coated with a PEG shell. Fourier transform infrared (FTIR) spectroscopy showed that the vibrational fingerprint of PEG around Au(50)Fe(50) NPs, collected by centrifugation after 30 days of incubation at 37 °C in FCS, totally disappeared, while the vibrational pattern of serum proteins, largely consisting of amide bands,^55^ is the only detectable one (Figure 3C). The characteristic peaks of PEG, such as the intense C–O–C stretching at 1100 cm^–1^, are observed in the samples before and after incubation for 1 h with FCS (collected by centrifugation), confirming the successful conjugation of the biocompatible polymer on the surface of the NPs. In fact, colloidal stability was assessed by dynamic light scattering (DLS, Figure S11 in SI) and evidenced that Au–Fe nanoalloys were stable in 20% v/v FCS even after 24 h of storage at 37 °C.

Since we verified that PEG bands are still visible in the physical mixtures with a large excess of bovine serum proteins (see Figure S12 in SI), FTIR spectroscopy suggests that the surface coating of the Au(50)Fe(50) NPs drastically changed during the incubation in FCS. The replacement of the stealth PEG coating with serum proteins during alloy degradation is a positive feature that may further facilitate the removal of NPs from the body fluids by sequestration in the MPS.^45,46,48^

### *In Vivo* Tests with Au–Fe NPs and Multimodal CA Ability

**Figure 4:**
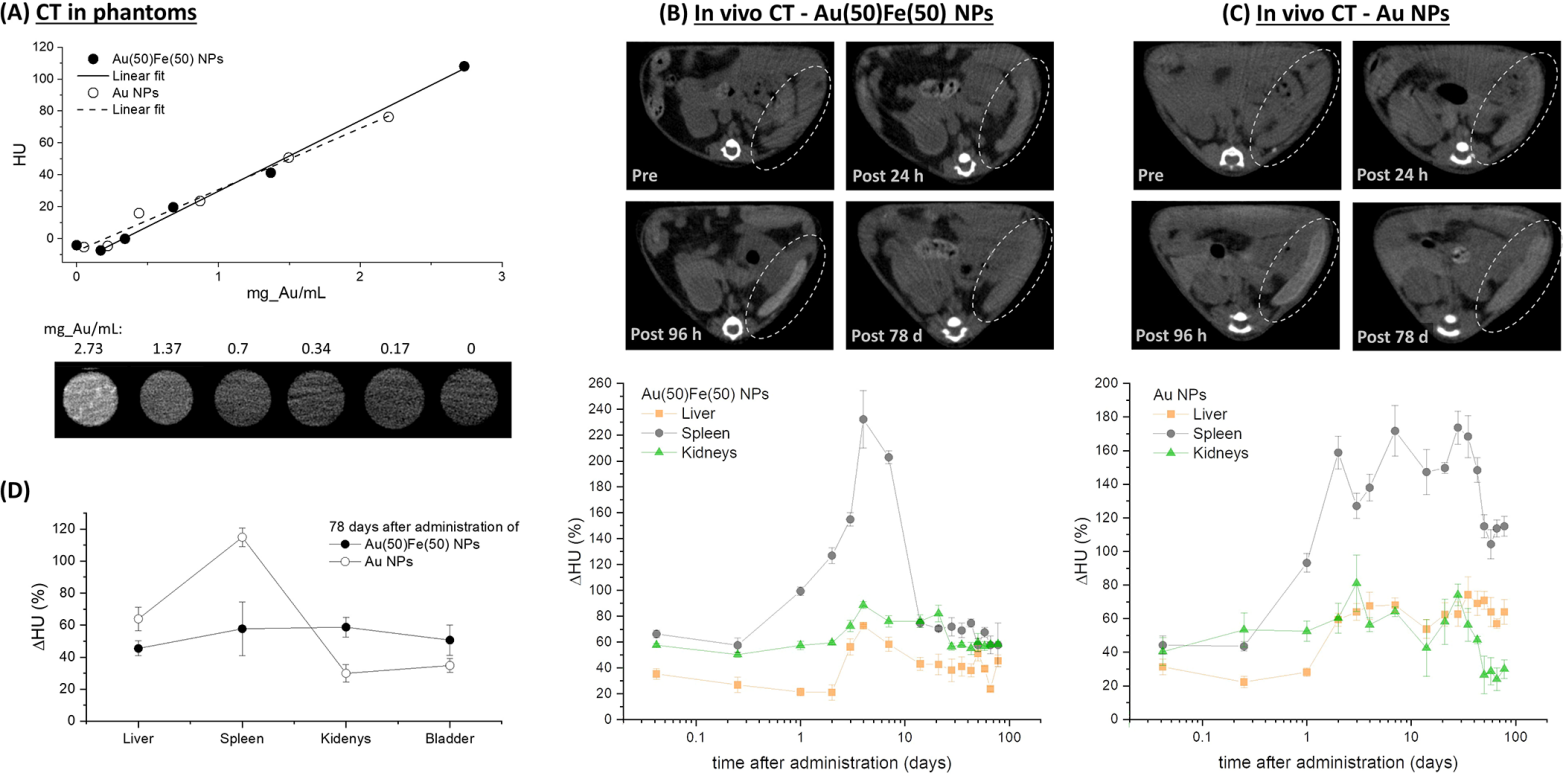
CT monitoring of the biodistribution. (A) Plot of HU *versus* Au concentration collected on phantoms containing the Au(50)Fe(50) NPs at variable dilution. CT images of phantoms’ cross-section are also reported. (B, C) Comparative biodistribution study of Au(50)Fe(50) and pure Au NPs administered on healthy mice and monitored by CT up to 78 days. Top: Images of mouse spleen showing the evolution of contrast over time, where it is appreciable that Au(50)Fe(50) NPs massively leave the spleen after 78 days, while Au NPs persisted to a large extent. Bottom: Plot of the relative increment of CT contrast over time, measured as ΔHU (%), in the liver, spleen, and kidneys. The biodistribution curve of Au(50)Fe(50) NPs exhibits a peak, while the curve of Au NPs shows a step, except for kidneys, which are not affected by Au distribution after 60 days. (D) Plot of ΔHU for liver, spleen, kidneys, and bladder at 78 days after administration, suggesting the flow of Au(50)Fe(50) NPs through the renal clearance pathway, in contrast to the persistence of Au NPs in the liver and spleen.

Next, the performances of the Au(50)Fe(50) NPs as multimodal CAs for CT and MRI were investigated. First, CT contrast ability was assessed in phantoms containing the Au(50)Fe(50) NPs in agarose gel at different concentrations (Figure 4A), and we measured the expected linearly increasing trend *versus* Au concentration. In the standard experimental conditions adopted (*i*.*e*., X-ray tube operating at 80 kV), a slope of 44 ± 2 HU mL/mg Au was measured, which is the same as found for a reference of pure Au NPs and higher than that reported in the literature for the commercially available iopromide (15.9 HU mL/mg) exploited in clinics.^21,56^ The measurements were then set up *in vivo*. Initially, the blood circulation and biodistribution of Au(50)Fe(50) NPs were examined by CT imaging of healthy mice injected intravenously with an NP dispersion in PBS (0.2 mL at 160 mg Au/kg body weight). Moderate CT signal enhancement (ΔHU = +5%) was measured in the mice’s brain 1 h after administration of the CAs, indicating the biodistribution in the blood. This is further confirmed by the comparable relative signal increment in the principal MPS organs (liver, spleen) and kidneys at the same time point (Figure 4B). A control experiment was performed, at parity of administered gold amount, with PEG-coated Au NPs, which are benchmark nanosized CAs. The pure Au CA showed the same biodistribution as the Au(50)Fe(50) NPs in the first hours after administration (Figure 4C). However, if the Au(50)Fe(50) NPs are degraded into small nanofragments, this should facilitate their excretion from the body compared to pure Au NPs. Hence, the contrast in major MPS organs and kidneys was monitored over time up to 78 days. As apparent from Figure 4B,C, the behavior of the two types of NPs is the same for the first 24 h, during which the CAs remain in the blood circulation. After 48 h, the contrast in liver and spleen increases steeply for the benchmark Au system, indicating sequestration of the NPs in these organs, while the ΔHU in kidneys remains stable at the initial level. This is reasonable for nondegradable CAs (PEG-coated Au NPs) with a blood circulation half-life on the order of 1–2 days. In fact, the ΔHU in the three organs remains constant up to 30 days; then it decreases from 174% to a plateau at 115% in the spleen and from 74% to a plateau at 30% in the kidneys at the last time point considered (78 days), but not in the liver (from 60% to 64%). Conversely, in the mice group treated with the Au(50)Fe(50) NPs, the ΔHU trend has a much more complex dynamics. The peaks in the contrast plots of liver and spleen are observed after 96 h instead of 48 h, suggesting a longer circulation time, and a peak is observed at 96 h also in the kidney plot. A longer circulation time, without immediate clogging of MPS organs or kidneys, is desirable to increase the chance of nanomedicine accumulation in lesions and malignant tissues for imaging or therapeutic purposes.^31,32^ The peak in the kidney curve further suggests that the particle size decreased over time up to matching the glomerular pore threshold, as expected for a degradable nanoparticle. In fact, the contrast decreases in all organs at times greater than 96 h, *i*.*e*., from 230% to 58% in the spleen, from 72% to 45% in the liver, and from 89% to 58% in the kidneys at the last time point considered. These values are comparable to those at 1 h after administration and lower than in the spleen and liver of the Au-treated mice (see Figure 4B,C). The decrease of ΔHU is delayed in kidneys compared to the liver and spleen, in agreement with the progressive degradation of the NPs into small fragments that are, in large part, cleared with urine over the 78 days of the experiment. In fact, a measurable contrast enhancement was found in the bladder of mice treated with the Au–Fe NPs (Figure 4D and S13). Noteworthy, after 78 days the contrast enhancement in the kidneys and bladder is comparable or larger than the spleen and liver for the mice treated with Au–Fe nanoalloys (Figure 4D), while it is much higher in the spleen and liver of Au-treated mice. This further suggests the extended degradation of Au–Fe nanoalloys into small fragments, which, according to previous observations on NPs less than 10 nm in size, undergo renal clearance through glomeruli filtration.^3,12^ Besides, in mice treated with the Au(50)Fe(50) NPs, the peak of the relative contrast increment in the spleen is at much higher values than in animals treated with Au NPs, while maximum signal increment remains comparable in the liver and kidneys for the two groups. This is compatible with the release of smaller NPs with high mobility and low interaction at the level of the hepatocytes in the liver, where only the largest objects are preferentially accumulated.^57,58^ It should be noted also that, when considering the mass of organs, NP accumulation in the kidneys is modest, confirming even more that these organs were not clogged by the degradable Au(50)Fe(50) NPs.

**Figure 5:**
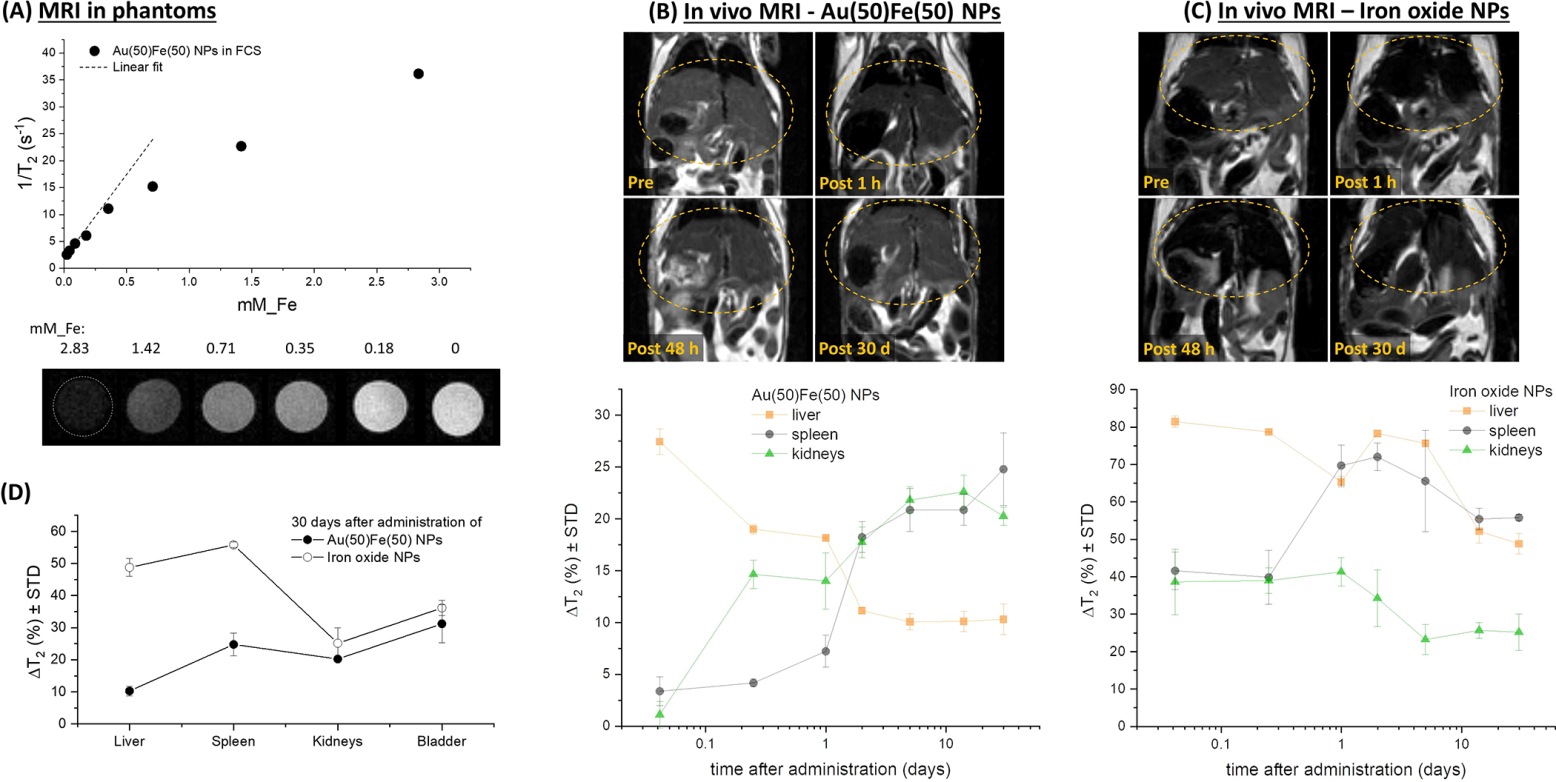
MRI monitoring of biodistribution. (A) Plot of relaxivity *versus* Fe concentration collected on phantoms containing the Au(50)Fe(50) sample at variable dilution. MRI images of phantoms’ cross-section are also reported. (B, C) Comparative biodistribution study of Au(50)Fe(50) and commercial iron oxide NPs (Endorem) administered on healthy mice and monitored by MRI up to 30 days. Top: Images of mouse liver showing the evolution of contrast over time, where it is appreciable that Au(50)Fe(50) NPs massively leave that organ after 30 days, while iron oxide NPs accumulate to a large extent. Bottom: Plot of the MRI contrast of over time, measured as the relative *T*_2_ signal intensity decrease (Δ*T*_2_, expressed in absolute % variation), in the liver, spleen, and kidneys. The biodistribution curves of Au(50)Fe(50) NPs exhibit a consistent decrease of Δ*T*_2_ in the liver and a sharp increase in the spleen and kidneys, while the curves of iron oxide NPs shows a slight decrease in the liver and kidneys and a sharp positive step in the spleen. (D) Plot of Δ*T*_2_ for liver, spleen, kidneys, and bladder at 30 days after administration, again suggesting the flow of Au(50)Fe(50) NPs through the renal clearance pathway, in contrast to the persistence of iron oxide NPs in the liver and spleen.

MRI contrast ability was assessed by measuring the transverse relaxation time (*T*_2_) of protons in water in a series of phantoms with variable concentration (Figure 5A), with a preclinical 7.0 T MRI scanner. In the low concentration range, the transversal relaxivity *r*_2_ scales linearly with iron molarity and is 31 ± 1 mM Fe^–1^ s^–1^, not far from the value of a benchmark commercial monomodal MRI CA based on iron oxide such as Endorem.^8,18^ However, it is worth emphasizing that CT CAs are used at a much higher concentration than MRI ones;^8,31,32,59^ hence the lower *r*_2_ is scarcely significant for an alloy functioning as a multimodal MRI/CT CA.

Then, the long-term fate of the Au(50)Fe(50) NPs was investigated in healthy mice by MRI (Figure 5B) and compared to a standard monomodal CA based on superparamagnetic iron oxide NPs (Endorem, Figure 5C) at parity of administered Fe content (5 mg Fe/kg body weight). MRI is essential to monitor over time the magnetic constituents of the nanoalloy, since iron is released during particle degradation and CT is sensitive only to Au biodistribution. As shown in the top of Figure 5B, a change in *T*_2_ was measured in the healthy mice at 1 h after intravenous administration of the CA dispersion in PBS, showing a clear contrast enhancement in the liver. The contrast decreased already after 48 h, until reaching a value close to the background after 30 days. This is better evidenced by the plot of *T*_2_ signal intensity decrease (Δ*T*_2_, expressed in absolute % variation) over time (bottom of Figure 5B), where signal dynamics has opposite trends in the liver than in the spleen and kidneys. The trend suggests that urine may be the dominant clearance pathway, as further corroborated by *T*_2_-weighted images of the bladder collected before and 30 days after administration of the CAs (Figure S14 in SI), where an appreciable contrast increment is found. Besides, the plot of Δ*T*_2_ after 30 days in the above-mentioned organs (Figure 5D) clearly shows that the NPs are moving from the liver and spleen toward the kidneys and bladder, as anticipated by the CT analysis.

The degradation of the Au(50)Fe(50) NPs is even more evident when comparing the MRI signal evolution with that of the iron oxide benchmark (Figure 5C). These dextran-coated iron oxide NPs provide a conspicuous contrast in MPS organs and kidneys already at the first time point, 1 h after administration, suggesting a massive sequestration. For longer times, the contrast remains comparable in all organs, although a decreasing trend is appreciable after the first 5 days. After 30 days (Figure 5C,D), the *T*_2_ signal variation is markedly higher in the spleen and liver than in the kidneys and bladder, as expected for biopersistent CAs. It is worth noting that, despite the biopersistence observed in this and previous studies,^18^ iron oxides are known as degradable nanomaterials recyclable in the endogenous blood iron pool,^3,15,16^ thus representing a reference for the realization of tolerable and clearable nanomedicine agents.

**Figure 6:**
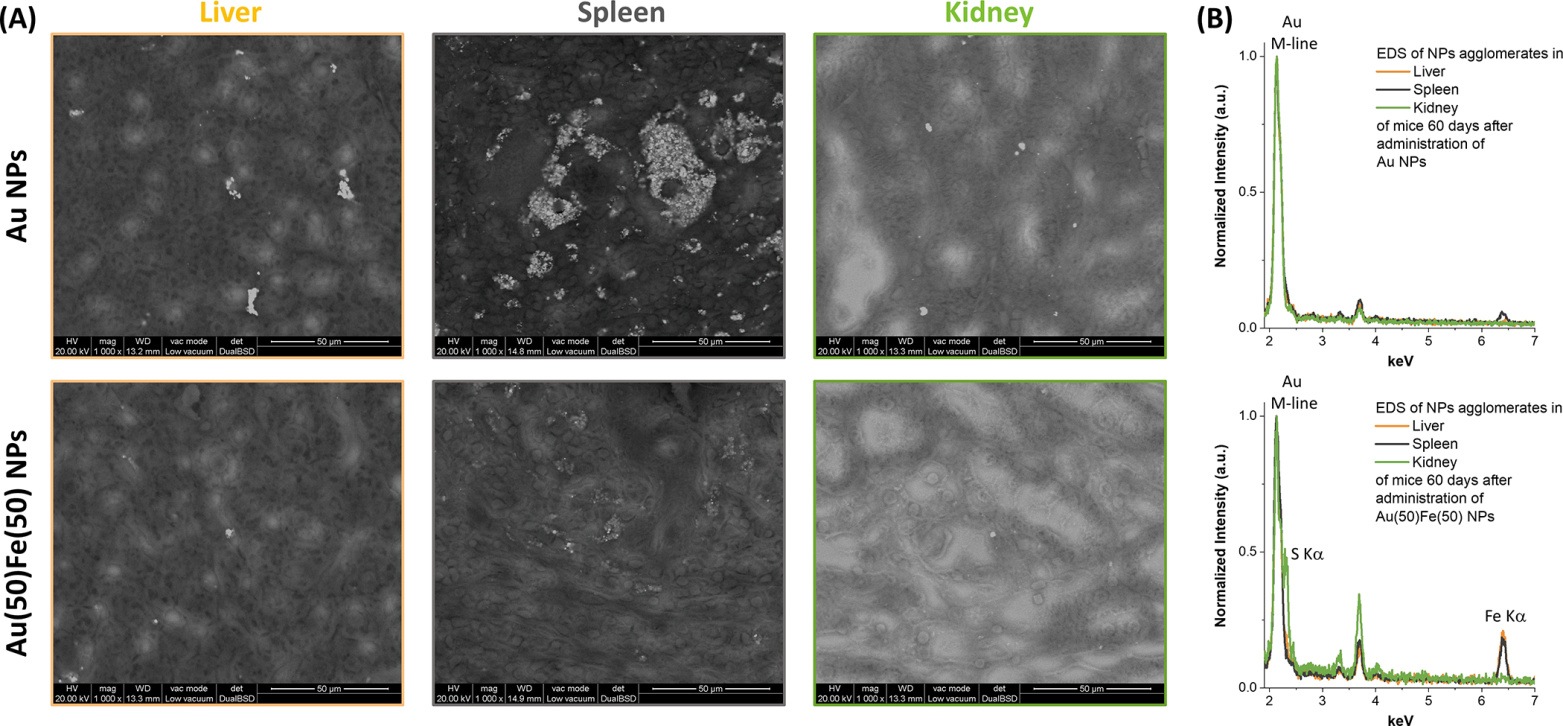
ESEM analysis of histopathological sections after 60 days. (A) Top: Mice treated with Au NPs. Large agglomerates of NPs are found in the spleen, and several clusters of NPs are found also in the liver and kidneys. Bottom: Mice treated with Au(50)Fe(50) NPs. Compared to the mouse treated with pure Au NPs, in this case there is a much lower density of NPs in all three organs. (B) EDS spectra collected on a group of NPs in each of the histopathological sections. The Au M-line peak is observed in all spectra, while the Fe Kα-line is found only in the liver and spleen of the mouse treated with the Au(50)Fe(50) NPs. In the kidney of the same animal, no Fe peak is detected, but the S Kα-line appears, suggesting that Au-rich nanoparticles coated with thiolated molecules reached this organ. Peaks in the 3–4 keV range belong to Ca and K.

Finally, the impact of Au(50)Fe(50) NPs on the main organs by long-time particle sequestration (*e*.*g*., liver, spleen, kidneys) was monitored by histopathological evaluation (Figure S15 in SI). The analysis revealed no microscopic lesions or alterations of cell morphology, and the tissues appeared as intact as in the animals treated with the reference PEG-coated Au NPs. This indicates that there is no evidence of toxic effects related to the release of iron species such as ferroptosis, which is an iron-concentration dependent effect,^60^ thus pointing to the excellent biocompatibility expected for a Au–Fe system and the exploitability of these nanoalloys for further theranostic applications. Besides, the investigation of the histopathological sections by environmental scanning electron microscopy (ESEM) evidenced the large accumulation of Au NPs in the spleen of the Au NP treated mice, apparent as sub-micrometric clusters, while the tissue of the Au(50)Fe(50) NP treated mice showed a sparse accumulation of small groups of NPs surrounded by an iron-rich region ascribable to the degradation of the alloy (Figure 6 and Figures S16 in SI). In agreement with the *in vivo* CT measurements (Figure 4) and the histopathological analysis (Figure S15), ESEM images indicate that also in the liver and kidneys the Au NPs are present with higher density than the Au(50)Fe(50) NPs (Figure 6A). However, the spleen appears as the main site of particle accumulation, as evidenced by the CT *in vivo* (Figure 4B,C). The EDS spectra collected on groups of NPs in each histopathological section (Figure 6B) allowed the identification of the Au M-line in all samples. Interestingly, the Fe Kα-line is found only in the Au(50)Fe(50) NPs in the liver and spleen, while it is absent in the kidney of the same animal. This suggests that alloy NP degradation occurs mainly in the spleen, while the small Au-rich fragments generated by the process are successively cleared through the kidneys with urine. In fact, histopathological analysis with iron-staining (S15 in SI) and ESEM images at higher magnification (S16 in SI) indicate the presence of an iron-rich region around each cluster of NPs, which is ascribable to the degradation of the alloy in the spleen. The same iron-rich region is not observed in the liver and kidneys of the same animal. Besides, the S Kα-line is detected in the EDS spectrum collected on the kidney of the mouse treated with the Au(50)Fe(50) NPs. This can be ascribed to the reaction of the small Au NP fragments generated by degradation of the initial Au(50)Fe(50) NPs with cysteine, homocysteine, cysteinylglycine, and glutathione present in the kidneys.^61^ NPs coated with these low molecular weight molecules can transit through the glomerular pores more easily than NPs coated with the more “bulky” serum proteins.

## Discussion

Altogether, our data demonstrate a dynamic evolution of the Au–Fe nanomedicine size and composition, in response to the appropriate environment and on a time scale that allows their use as CAs for CT and MRI, which are the most common and effective noninvasive medical imaging techniques,^31,32^ before structural degradation triggers body clearance. For clinical applications, it is important that CAs are safely eliminated from the body after performing their functions.^7,31,32^ Monomodal molecular CAs are cleared by the organism in a few hours through the kidneys.^12^ For instance, 40% of 2.5 nm Au NPs injected in mice were cleared after only 24 h in the urine, while a non-negligible accumulation in the liver and spleen of the animals also occurred, which gradually decreased over 1 month after injection.^62^ This result agrees with the observation that the size threshold for glomerular filtration is inhomogeneous and extends up to several tens of nm and that in any filtration process small objects pass more rapidly that large objects.^63^ In the case of ultrasmall (nano)drugs, this forces the administration of high doses, which also implies high risks of adverse reactions such as anaphylactic shock, renal impairment, and syndromes such as nephrogenic systemic fibrosis induced by Gd-based CAs.^31,32^ Nano-CAs have the opposite problem of massive sequestration by the MPS and prolonged biopersistence,^3,8,31,32^ which motivated the large number of studies on inherently biocompatible nanostructures based on iron and gold.^3,7,15,16,64^ Nonetheless, it is well known that iron compounds, typically oxides, and gold NPs also lead to concerns of long-term toxicity.^31,32,45,48^ The combination of iron oxides and gold in a single nanomedicine agent does not represent an exception.^8,9^ In NPs composed of a Au shell surrounding an iron oxide core, a complete shielding of the core against the harsh lysosomal environment was observed inside cells.^13,30^ This is in agreement with our calculations of diffusion barriers for Fe and O atoms through the dense fcc lattice of Au, shown in Figure 1B. Only when the gold layer is thin and porous does corrosion of the oxide core proceed beneath the shell.^30^ In fact, the degradation of iron oxide compounds relies on the interaction with the several species present in physiological environment, including enzymes and chelating agents necessary to degrade the exogenous particles, which are brought by lysosomes and endosomes.^19,20^

Similarly, the degradation of Au–Fe alloy is believed to be a step-by-step corrosion process governed by surface reaction mechanisms occurring in two stages: oxidation of metal iron to iron oxide and cleavage from the particle surface by chelating compounds.^15,20^ The thermodynamic driving force to oxidation of metallic Fe is not affected by alloying with Au, as it was quantitatively shown by free energy calculations reported in Figure 1A. Successively, the iron-chelating groups dispersed in physiological and lysosomal environments bind with oxidized Fe atoms, leading to the cleavage of the Fe–O bonds^16^ and the degradation of Au–Fe nanoalloys. Then, corrosion proceeds by etching the bulk of the alloy NP by attacking along randomly distributed regions of high defect densities in the particle interior and forming small cavities.^37,52,53,65^ During the etching process, these cavities gradually grow and coalesce, until fragmentation of the pristine Au–Fe alloy into smaller gold-rich fragments.^30^

It is thus clear that there are two main mechanisms through which the NP architecture affects the degradation process. The first mechanism that provides self-degradation properties to the alloy is the presence of iron percolation paths throughout the metal lattice, where corrosion can proceed. In general, these paths are a function of alloy composition,^34,35,38^ as shown in Figure 1E. However, enthalpy-weighted simulations of alloy formation evidenced how element topology is more important than bare stoichiometry in defining the compositional threshold for the appearance of the percolative paths. In the case of nonequilibrium alloys such as the Au–Fe system, due to the tendency of Au and Fe to separate, this was calculated already at 43 at. % of Fe or even less, depending on the thermal disorder parameter τ.

Finally, it is well recognized that polymer layers play a relevant protective role in iron oxide compounds.^15,20^ Therefore, the use of a thiolated PEG shell to stabilize the Au–Fe NPs is the second important feature for conferring the self-degradation behavior. In fact, thiols bind to Au atoms, leaving surface Fe atoms free from any organic coating that prevents dissolution, as happened in previous studies with polymer-coated iron oxides^15,19^ and in this study with Endorem. The replacement of the PEG shell with serum proteins upon alloy dissolution, as indicated by the FTIR analysis of Figure 3C, further confirms the surface accessibility of the NPs as part of the 4D evolution.

The use of a simple, green, and low-cost synthetic route such as laser ablation in liquid, which bypasses thermodynamic limitations to the fabrication of Au–Fe NPs and provides complete flexibility in surface coating with the desired thiolate compound,^33,42^ is thus crucial to achieve 4D nanomedicines.

## Conclusions

In summary, we showed how to develop 4D nanomedicines based on nonequilibrium Au–Fe alloys, and we evidenced a series of positive features for use of these NPs as a degradable multimodal CA for combined MRI and CT imaging. The 4D behavior allows the self-degradation of the Au–Fe NPs in physiological environment at basic and acidic pH, giving smaller Au-rich NPs over several days, which is desirable to facilitate the clearance of administered nanomedicines. Self-degradation can be triggered also by external chemical stimuli, such as the addition of EDTA. Key for alloy degradation is the presence of percolative Fe paths inside the NPs. DFT calculations and mixing enthalpy-weighted alloying simulations evidenced that the Fe percolation threshold is lower in the nonequilibrium Au–Fe system compared to thermodynamically permitted bimetallic alloys, because of its tendency to segregate elements combined with impaired atomic diffusivity. We believe that these Au–Fe nanoalloys, featuring bimodal CT/MRI CAs performance, self-degradation, and facilitated clearance from the body, hold great promise for the development of next-generation nanomedicines. One would envision the use of the 4D alloys in multiple biomedical applications, such as the improvement of lesion detection using noninvasive combined imaging. Therefore, this research provides a general approach to realize on-demand biodegradable inorganic theranostic agents, which is expected to help solve the critical low degradation issue of inorganic nanomedicines.

## Materials and Methods

### Computational Modeling

DFT calculations were performed using the plane-wave pseudopotential approach, as implemented in Quantum-ESPRESSO,^66^ where the PBE^67^ approximation to the exchange–correlation functional was employed alongside the pseudopotentials from the GBRV library.^68^ The cutoff on wave functions was 30 Ry in slab and NEB calculations and 35 Ry in variable-cell optimization of special quasi-random structures (SQSs), while the cutoff on charge density was 300 Ry in all cases. Slab models were composed of five atomic layers of metals, and the bottom two layers remained frozen in their bulk positions. Water molecules, OH, and O were adsorbed only on the top side of the slab, and periodic replicas along the *z* direction were separated by a vacuum of width larger than 10 Å.

Topological analyses of percolation paths on large alloy models were performed using a homemade code written in Fortran90 and parallelized using the MPI paradigm.

QE inputs for DFT calculations and inputs and codes for numerical simulations on percolation paths are available from the authors (D.F.) upon reasonable request.

### Synthesis and Characterization

NPs were synthesized by laser ablation in liquid, according to a modification of a previously established procedure.^42^ Metal targets (Au, Au/Fe 75/25 at. %, Au/Fe 50/50 at. %, Au/Fe 25/75 at. %, >99.99% pure, from Mateck GmBH) were placed at the bottom of a cell containing PEG-SH (5000 Da, Laysan Bio) 0.085 mg/mL in ethanol (HPLC grade, from Sigma-Aldrich) and ablated with laser pulses (1064 nm, 6 ns, 50 Hz) focused at a fluence of 18 J/cm^2^ by a lens with focal length *f* = 100 mm. The colloid was then stored at −20 °C overnight, collected by centrifugation at 1000 rcf for 45 min at 5 °C, and washed three times with methanol and ethanol by centrifugation at 1000 rcf for 45 min at 5 °C to remove unbound PEG-SH and any other synthesis byproduct. Finally, the NPs were dried and resuspended in the desired aqueous solution. All chemicals were used without further purification.

Elemental analysis to assess sample elemental composition and concentration was performed by ICP-MS with an Agilent Technologies 7700x ICP-MS (Agilent Technologies International Japan, Ltd., Tokyo, Japan). The instrument is equipped with an octupole collision cell operating in kinetic energy discrimination mode, which was used for the removal of polyatomic interferences and argon-based interferences. The multielement calibration standard-3 (Agilent Technologies) for Au and CLPP-CAL-1 (Inorganic Ventures’ Calibration Standard 1) for Fe were used. Multielement standard solutions for calibration were prepared in 5% aqua regia by gravimetric serial dilution at six different concentrations (from 0.5 to 1000 μg L^–1^). A microwave acidic digestion was performed with a CEM EXPLORER SP-D PLUS.

XRD patterns were collected from powder samples deposited on Si zero-background substrates with a Panalytical XPert 3 powder diffractometer equipped with a Cu tube (40 kV, 40 mA), a BBHD mirror, a spinner, and a PlXcel detector. Crystalline phase identification and Rietveld analysis were executed with the Panalytical High Score Plus 4 software and Panalytical ICSD, PDF2, and COD databases. NPs were analyzed as obtained from the synthetic procedure or after 2 months’ incubation at 37 °C and 0.15 mg/mL concentration in PBS (pH 7.4) or citrate buffer (pH 4.7) solutions, by collecting the lyophilized samples. Optical absorption spectroscopy was performed with a JASCO V770 UV–vis–NIR spectrometer in 2 mm quartz cells on the same samples in PBS and citrate buffers used for XRD analysis, at time points 0 and after 2 months.

SAXS was performed on an XEUSS 1.0 instrument from XENOCS, equipped with a Cu Kα microfocus source (λ = 0.154 18 nm) and PILATUS-100 K detector. Sample-to-detector distance was kept at 1350 mm spanning a *q*-range from 0.08 to 2.6 1/nm. The samples were injected in borosilicate glass capillary tubes of 1.5 mm in diameter and 10 μm in wall thickness. A one-dimensional pattern was obtained by integration of the 2D data using the Foxtrot program.^69^ Nanoparticles were dispersed in Milli-Q water at 0.2 mg/mL concentration, stored at 37 °C, and analyzed at time points of 0 and 60 days.

Specific magnetization (*M*) as a function of applied magnetic field (*H*) at room temperature was obtained using a vibrating sample magnetometer (LakeShore 7404), operated with maximum applied fields μ_0_*H*_max_ = 1.5 T. Magnetic measurements were performed on the NP samples dispersed in Milli-Q water. Each colloid suspension was sealed into a heat-shrinkable tube to prevent sample evaporation and spills.

Bright field TEM analysis was performed with a FEI Tecnai G2 12 operating at 100 kV and equipped with a TVIPS CCD camera. NPs were dispersed at 0.035 mg/mL concentration in 20% v/v FCS at pH 7.4 or 4.7 (by adding citrate buffer), incubated at 37 °C, and analyzed at different time points from 0 h to up to 1440 h (2 months). Each time, a drop of the solution was deposited on a copper grid coated with an amorphous carbon film. Statistics considered more than 500 NPs for each sample, using the ImageJ software. The experiment for the quantification of dissolved metal atoms at different incubation times by ICP-MS was performed after separation of the liquid solution from serum proteins and nanoparticles by dialysis (Sartorius 3 kDa concentration membranes).

HRTEM and STEM analyses were performed with a TEM Talos F200S G2.

In the experiment of NP degradation triggered by a chemical compound, Au(50)Fe(50) NPs were dispersed at 0.035 mg/mL concentration in 20% v/v FCS with 0.33 mg/mL EDTA and incubated at 37 °C for 4 h prior to TEM analysis.

FTIR of the powder samples deposited on a KBr window were collected with a PerkinElmer 1720X spectrometer. Au(50)Fe(50) NPs were analyzed as obtained from the synthetic procedure and after 1 h and 1 month incubation at 37 °C and 0.035 mg/mL concentration in 20% v/v FCS. In the latter case, NPs were collected by centrifugation at 30 000 rcf for 3 h at 18 °C, washed with deionized water three times at 30000 rcf for 3 h at 18 °C, and finally dried for deposition on the KBr window.

DLS was performed with a Malvern Zetasizer Nano ZS on Au(50)Fe(50) samples dispersed in 20% v/v FCS at 0.035 mg/mL.

### CT Experiments

CT images were acquired using a dedicated small-animal CT scanner (x-rad, SmART, Precision X-ray) using the following acquisition parameters: tube tension 80 kVp, current 3 mA, 300 views, 0.1 mm voxel size. Images were reconstructed using the Feldkamp algorithm for cone beam CT. In phantom measurements, the samples were dispersed in 1% agarose solution by serial dilutions, starting from a concentration of 2.73 mg Au/mL. X-ray attenuation ability in HU mL/mg Au was calculated from the slopes of the best fit lines of HU *versus* gold concentration.

To evaluate *in vivo* the biodistribution of Au or Au(50)Fe(50) NPs, Balb/c male mice (10 weeks old) were intravenously injected with NPs at a dosage of 160 mg Au/kg (200 μL) in the mouse tail vein. CT image acquisitions *in vivo* were performed before and at 1 h, 6 h, 24 h, 48 h, 72 h, 96 h, 7 days, 14 days, 21 days, 28 days, 35 days, 43 days, 50 days, 58 days, 66 days, and 78 days after injection. During image acquisition, the animals were kept at 37 °C and under gaseous anesthesia (2–3% isoflurane and 1 l/min oxygen). No weight loss or signs of suffering were detected in mice over the 2 months of the *in vivo* experiment. Image analysis (using imageJ) was performed by placing five different region of interest (ROI) on the corresponding organ (liver, spleen, kidneys, bladder), and the mean HU value of each ROI was calculated.

For the histopathological assessment, formalin-fixed paraffin-embedded consecutive sections (4 μm) were dewaxed and hydrated through a graded decreasing alcohol series and stained for histological evaluation in bright field microscopy. Slides were stained using standard protocols for hematoxylin and eosin (using Mayer’s hematoxylin, BioOptica #05-06002/L, and eosin, BioOptica #05-10002/L) and iron stain (Abcam, ab150674). SEM-EDX analysis was performed with a FEI Quanta 200 environmental SEM without any sample metallization.

### MRI Experiments

Magnetic resonance images were acquired using a Bruker system operating at 7 T (Bruker Biospin, Ettlingen, Germany). In phantom measurements, the samples were dispersed in aqueous solution by serial dilution starting from a solution with an Fe concentration of 2.83 mM. The transversal relaxation times (*r*_2_ value) were calculated from the slopes of the best fit lines of relaxation rates (1/*T*_2_) *versus* iron concentrations. The *T*_2_ map phantom images were acquired using a multislice multiecho sequence with the following parameters: TR = 2000 ms, TE = from 6.5 to 170.43 ms, FOV = 55 × 55 mm, matrix size = 128 × 128, slice thickness = 1 mm, number of echoes = 25.

To evaluate *in vivo* the biodistribution of Au(50)Fe(50) NPs and of benchmark Endorem, Balb/c male mice (6–8 weeks old, Envigo) were intravenously injected with NPs at a dosage of 5 mg Fe/kg. For MRI acquisitions, animals were anesthetized with gas anesthesia (a mixture of O_2_ and air containing 1–1.5% isofluorane), placed in a heated animal bed, and inserted in a 7.2 cm internal diameter bird-cage coil. *T*_2_-weighted images of the mouse body were acquired using a rapid acquisition with relaxation enhancement (RARE) sequence with the following parameters: FOV = 60 × 40 mm, MTX = 256 × 256, slice thickness = 1 mm, TE = 33 ms, and TR = 2.500 ms. The images were acquired before and 1 h, 24 h, 48 h, 5 d, 2 weeks, and 1 month after NP injection.

## Supporting Information Available

Computational details on surface models, surface reactivity toward H_2_O and O_2_, bulk diffusion, bulk models, topological model of dealloying; additional experimental data on structural characterization and *in vivo* NP biodistribution (PDF)
