# Supplementary material for: 4D Multimodal Nanomedicines Made of Nonequilibrium Au–Fe Alloy Nanoparticles
Source: ACS Nano. 2020 Sep 2;14(10):12840–53. doi: 10.1021/acsnano.0c03614 (PMC8011985; doi:10.1021/acsnano.0c03614)
Supplement: Supplementary file 1 — nn0c03614_si_001.pdf [file nn0c03614_si_001.pdf]

Supporting Information

4D Multimodal Nanomedicines Made of Non-Equilibrium Au-Fe Alloy Nanoparticles

Veronica Torresan,[1,§] Daniel Forrer,[1,2,§] Andrea Guadagnini,[1] Denis Badocco,[1] Paolo Pastore,[1] Maurizio Casarin,[1,2] Annabella Selloni,[3] Diego Coral,[4] Marcelo Ceolin,[5] Marcela B. Fernández van Raap,[6] Alice Busato,[7] Pasquina Marzola,[7] Antonello E. Spinelli,[8] Vincenzo Amendola[1,*]

[1] Department of Chemical Sciences, University of Padova, Padova, I-35131 Italy
[2] CNR – ICMATE, Padova, I-35131 Italy
[3] Department of Chemistry, Princeton University, New Jersey, 08544 United States
[4] Departamento de Fisica, Universidad del Cauca, 193577 Popayán-Colombia
[5] Instituto de Investigaciones Fisicoquımicas Teoricas y Aplicadas (INIFTA), Departamento de Quımica, Facultad de Ciencias Exactas, Universidad Nacional de La Plata – CONICET, La Plata, 1900 Argentina
[6] Instituto de Física La Plata (IFLP), Departamento de Física, Facultad de Ciencias Exactas, Universidad Nacional de La Plata – CONICET, La Plata, 1900 Argentina
[7] Department of Computer Science, University of Verona, Verona, 37134 Italy
[8] Experimental Imaging Centre, IRCCS San Raffaele Scientific Institute, Milan, 20132 Italy

[§] Authors with equal contributions

* correspondence: vincenzo.amendola@unipd.it

Contents: *page*

Computational Details S2
S1. Surface models S2
S2. Surface reactivity towards H_2O and O_2 S3
S3. Bulk diffusion S5
S4. Bulk models S7
S5. Topological model of dealloying: S9
Experimental data S12
S6. Additional structural characterization data S12
S7. Additional *in vivo* biodistribution data S17
Bibliography S20

Computational Details

Calculations were performed with Quantum-Espresso [1], using the PBE [2] approximation to the xc functional and ultrasoft pseudopotentials from the GBRV library [3]. We used a plane-wave cutoff of 30 Ry and a cutoff on the density of 300 Ry. The cutoff on plane waves was increased to 35 Ry in variable-cell calculations, to reduce Pulay stress[4].

S1. Surface models

Given the size of the AuFe NPs under study, we model their surfaces using a periodic slab approach and, since Au NPs typically expose (1 1 1) surfaces, we restrict ourselves to the modeling of such a low-index surface. Water and hydroxide form a $\sqrt{3} \times \sqrt{3}R30°$ structure on fcc (1 1 1) metal surfaces, with 2 water molecules for every 3 surface atoms [5], therefore we chose two surface cells able to reproduce such structures, namely a $\sqrt{3} \times \sqrt{3}R30°$ surface cell with 3 atoms in the top layer and a $\sqrt{3} \times 2\sqrt{3}R30°$ having 6 surface atoms. All slab models contain 5 metal atomic layers, the bottom 2 of which are kept frozen in their bulk positions, molecules are adsorbed on the top side only and adjacent slabs along the direction normal to the surface are separated by a vacuum of width larger than 10Å. Results are collected in table S1 and selected slab models are shown in figure S1.

In all cases, Fe is found to be more stable at the 3rd layer rather than at the surface. This is in tune with previous calculations [6] and is also in agreement with the observation that Au has a lower surface energy than Fe[7].

Table S1: relative stability of AuFe slab models for different positions of the Fe atoms. Energy values are given per $\sqrt{3} \times \sqrt{3}R30°$ cell.

	Relative stability (eV)		
model	surface	subsurface	3rd layer
$\sqrt{3} \times 2\sqrt{3}R30°$<br>16.7% Fe in layer	0.24	0.01	0
$\sqrt{3} \times \sqrt{3}R30°$<br>33% Fe in layer	0.50	0.00	0
$\sqrt{3} \times \sqrt{3}R30°$<br>66.7% Fe in layer	1.23	0.02	0
$\sqrt{3} \times \sqrt{3}R30°$<br>100% Fe in layer	1.64	0.17	0

The presence of adsorbed water does not change this picture: Fe at the surface is always disfavored (Table S2). While the adsorption energy of water is larger when it interacts directly with Fe atoms, such a gain in energy does not compensate for the lower stability of Fe at the surface. The presence of Fe in the subsurface does not significantly modify the interaction of water with Au surface atoms in comparison to a pure Au slab. For the latter, the adsorption energy of a single water molecule is -0.17 eV and the adsorption energy of a full monolayer is -0.50 eV per molecule.

Figure S1: a) Top and b) side views of the modelled water monolayer adsorbed on the Au (1 1 1) surface. c) Top and d) side views of a partially dissociated water monolayer, corresponding to $*-OH$ in text. White solid lines depict the $\sqrt{3} \times \sqrt{3}R30°$ surface cell, while the $\sqrt{3} \times 2\sqrt{3}R30°$ cell includes also the portion in dashed lines. Color code: yellow: Au; bright red: Fe; dark red: O; bright cyan: H.

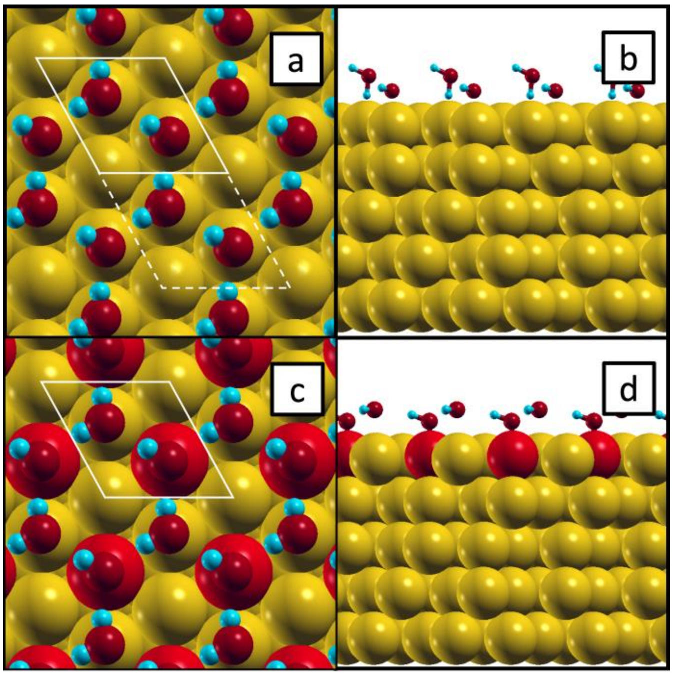

Table S2: Adsorption energies E_{ads} of water per H_2O unit and relative stabilities per $\sqrt{3} \times \sqrt{3}R30°$ cell of AuFe slab. Slab models have a $\sqrt{3} \times \sqrt{3}R30°$ surface cell and contain different amount of Fe atoms located either at the surface of at the subsurface layer.

	E_{ads} (eV) per H_2O		*Relative stability (eV)*	
	Fe@surface	*Fe@subsurface*	*Fe@surface*	*Fe@subsurface*
Fraction of Fe at layer	**1 H_2O molecule**			
1/3	-0.50	-0.18	0.15	0
2/3	-0.68	-0.15	0.67	0
3/3	-0.46	-0.20	1.20	0
	**H_2O monolayer**			
1/3	-0.62	-0.50	0.23	0
2/3	-0.60	-0.47	0.95	0
3/3	-0.56	-0.50	1.33	0

S2. Surface reactivity towards H_2O and O_2

For dealloying to happen, Fe must be oxidized at the interface with water. Such an oxidation process can be caused by either water itself or by atmospheric oxygen dissolved therein. To verify whether Fe can be oxidized even when embedded in an Au matrix, we investigated the following reactions:

$$* -OH_2 \rightarrow * -OH + \frac{1}{2}H_2 \quad (1)$$

$$* -OH_2 \rightarrow * -O + H_2 \quad (2)$$

$$* - OH_2 + \frac{1}{2} O_2 \rightarrow \ * - O + H_2O \qquad (3)$$

Here, $*$ represents a Fe surface site and in all cases the starting point is the hydrated surface.

For each process the reaction free energy is computed as

$$\Delta_r G = \Delta_r U + \Delta(PV) - T\Delta S$$

where $\Delta_r U$ is the difference in total energy between products and reactants computed at the DFT level $\Delta_r E_{DFT}$, plus the change in Zero-Point Energy (ZPE)

$$\Delta_r U = \Delta_r E_{DFT} + \Delta ZPE$$

ZPE values are taken from literature data [8,9] referring to adsorbates on Cu and Pt surfaces. Similarly with what has been done in other works [8], we assume that the major contribution to ZPE changes would be independent on the actual nature of metal under investigation. The $\Delta(PV)$ term is evaluated within the ideal gas approximation and standard entropies are taken from thermodynamic tables[10].

The reference state of molecules was chosen to fit as much as possible experimental conditions: the water reference state is the gas phase at P = 0.035 bar and T = 298.15 K, because at that pressure water vapor is in equilibrium with its liquid phase; oxygen reference state is again the gas phase at the partial pressure of 0.21 bar, its typical partial pressure in the atmosphere which is in equilibrium with the water medium; for the same reason, hydrogen reference state is the gas phase at the partial pressure of $5.6 \times 10^{-7}\ bar$. Absolute entropies of the molecular species were therefore calculated as $S = S° - k_B \ln(P_i)$, where $S°$ is the standard entropy, k_B is the Boltzmann constant and P_i is the partial pressure. Roto-translational motion of adsorbed species is frozen and their entropy is small compared to either gas phase or liquids, then the entropy of adsorbed species was neglected.

Surfaces were built using four different alloy models, namely a $\sqrt{3} \times \sqrt{3}R30°$ surface model with either 2 or 3 (out of 3) Fe at the surface and a $\sqrt{3} \times 2\sqrt{3}R30°$ having either 1 or 2 (out of 6) Fe atoms in the top layer. All models are covered with a water monolayer similar to the one shown in figure S1a-b and reactions involve one H_2O unit directly bonded to a surface Fe. Results are collected in table S3.

Oxidation of Fe by water with formation of surface hydroxide is always favored, whereas the formation of surface oxide is possible only when the amount of Fe is large enough. Conversely, formation of oxide through reaction with molecular oxygen is always favored.

Table S3: Reaction energies $\Delta_r E_{DFT}$, Zero-Point energy change ΔZPE, $-T\Delta S$ and free Gibbs energies $\Delta_r G$ of three oxidation processes, as a function of the atomic fraction of Fe at the surface.

Process:	Fe Fraction at surface	$\Delta_r E_{DFT}\ (eV)$	$\Delta ZPE\ (eV)$	$-T\Delta S\ (eV)$ T=298.15 K	$\Delta_r G\ (eV)$
$* - OH_2 \rightarrow \ * - OH + \frac{1}{2} H_2$	1/6	0.14	-0.23	-0.20	-0.28
	2/6	0.17			-0.25
	2/3	-0.49			-0.91
	3/3	-0.43			-0.84
$* - OH_2 \rightarrow \ * - O + H_2$	1/6	1.40	-0.32	-0.77	0.32
	2/6	1.02			-0.05
	2/3	0.62			-0.45
	3/3	0.27			-0.81

$* - OH_2 + \frac{1}{2} O_2 \rightarrow * - O + H_2O$	1/6	-1.10	-0.83	-0.33	-1.42
	2/6	-1.48			-1.79
	2/3	-1.88			-2.19
	3/3	-2.23			-2.55

S3. Bulk diffusion

The diffusion of Fe and Au atoms in the bulk alloy is relevant for understanding the aging of NPs and for identifying a possible corrosion mechanism. We investigated this process by means of Nudged Elastic Band (NEB)[11] calculations on 2x2x2 fcc conventional supercells containing 32 sites. We considered two distinct mechanisms for the diffusion of metal atoms: a collective movement of 3 atoms in the perfect fcc lattice, and a diffusion through vacancies. Results are collected in table S4. Unsurprisingly, the diffusion through vacancies is favored over the ring mechanism in both the case of Au self-diffusing in an Au matrix and of Fe diffusing in the Au lattice [12]. Duhl *et al.* [13] provide an experimental reference for the activation energy barriers, namely 1.83 eV for Au self-diffusion and 1.80 eV for Fe diffusion in Au bulk. These values can be compared to the sum of the Gibbs free energy of formation of an Au vacancy plus the free Gibbs energy barrier, which we estimated as the sum of proper DFT energies, thus assuming that changes in zero-point energy (ZPE) and entropy are negligible. In both cases the computed values underestimate experimental ones. Indeed more sophisticated elaboration would be needed to quantitatively predict these values [14], which is however beyond the scope of the present work. Still, we can infer that diffusion of either Fe or Au is frozen at room temperature.

The fcc lattice can be seen as composed by tetrahedral and octahedral voids, with each octahedron sharing its faces with tetrahedrons and vice versa. Oxygen was found to prefer tetrahedral voids in most cases. In pure Au the tetrahedral interstice is 0.48 eV more stable than the octahedral one, whereas this difference drops to 0.24 eV when one vertex is Fe.

O atoms shows a fair mobility in Au bulk (E_a = 0.58eV), which decreases rapidly as Fe is introduced in the lattice. Due to the larger affinity of oxygen towards Fe, O atoms easily attach to Fe impurities and can move around the Fe atoms easily, indeed the diffusion of oxygen from an octahedral void sharing a Fe vertex is almost barrierless, while the movement in the opposite direction is only 0.24 eV. The breaking of the FeO bond shows a barrier of 1.54eV, meaning that the strong bonding with Fe will prevent the diffusion of oxygen through pure Au regions and then the presence of an Au shield will passivate the alloy against passivation. As the number of Fe atoms increases, the diffusion of O along the Fe pattern increases, showing that O diffusion will be very slow at room temperature.

Table S4: Energy changes (ΔE) and activation energies E_a (in eV) for the diffusion of relevant species in pure Au and in dilute AuFe alloy. In case of vacancy diffusion, formation energies of vacancies (E_{vac}) are also reported. Blue diamonds and orange triangles represent octahedral and tetrahedral voids, respectively.

Process	Model		Energies
Au self-diffusion in bulk	through ring mechanism	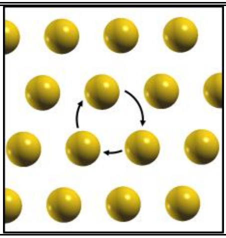	ΔE = 0 eV<br>E_a = 4.73 eV

	through vacancy	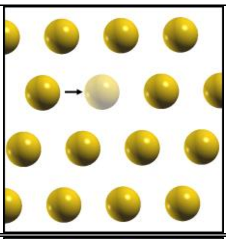	$\Delta E = 0$ eV<br>$E_a = 0.58$ eV<br>$E_{vac} = 0.44$ eV
Iron diffusion in bulk Au	through ring mechanism	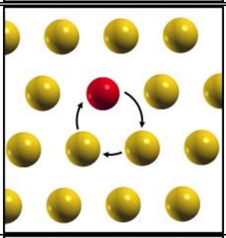	$\Delta E = 0$ eV<br>$E_a = 3.04$ eV
	through vacancy	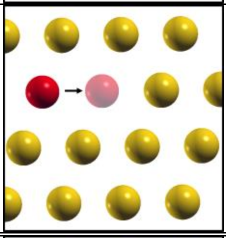	$\Delta E = 0$ eV<br>$E_a = 0.71$ eV<br>$E_{vac} = 0.49$ eV
Oxygen diffusion in bulk Au	octahedral to tetrahedral site	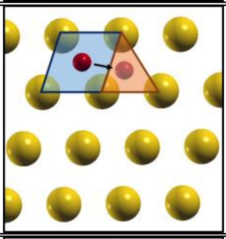	$\Delta E = -0.48$ eV<br>$E_{a->} = 0.10$ eV<br>$E_{a<-} = 0.58$ eV
Oxygen diffusion in AuFe alloy	around Fe atom in Au bulk, octahedral to tetrahedral site	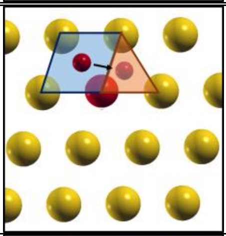	$\Delta E = -0.24$ eV<br>$E_{a->} = 0.00(4)$ eV<br>$E_{a<-} = 0.24$ eV
	towards Fe in Au bulk, octahedral to tetrahedral site	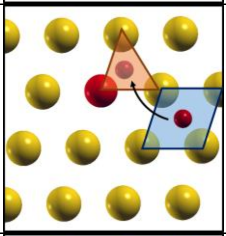	$\Delta E = -1.13$ eV<br>$E_{a->} = 0.41$ eV<br>$E_{a<-} = 1.54$ eV
	along a 2 Fe atoms path in Au bulk, tetrahedral to octahedral site	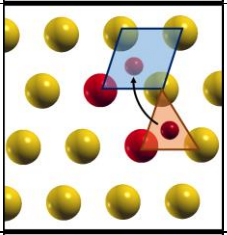	$\Delta E = -0.84$ eV<br>$E_{a->} = 0.53$ eV<br>$E_{a<-} = 1.37$ eV
	along a Fe 3-atoms path in Au bulk, tetrahedral to tetrahedral site	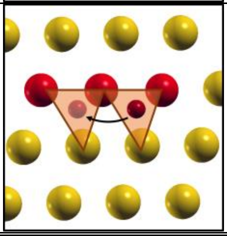	$\Delta E = 0$ eV<br>$E_{a->} = 1.68$ eV

S4. Bulk models

The AuFe alloy shows a fcc crystal structure over a broad range of compositions, with Au and Fe atoms randomly distributed among the lattice sites. Therefore, we use special models of the alloy (Figure S2), known as Quasi-Random Structures (SQS), having the special property of reproducing its long-range correlation function. These models contain 32 atoms, *i.e.* 8 fcc conventional cells, and are taken from ref [15], where they have been successfully used to describe the elastic properties of binary alloys with fcc structure.

Figure S2: Special Quasi-Random Structures of the fcc binary alloy [15]. Red atoms are Fe and yellow atoms are Au. Atomic positions are optimized, while the cell is kept fixed as described in text.

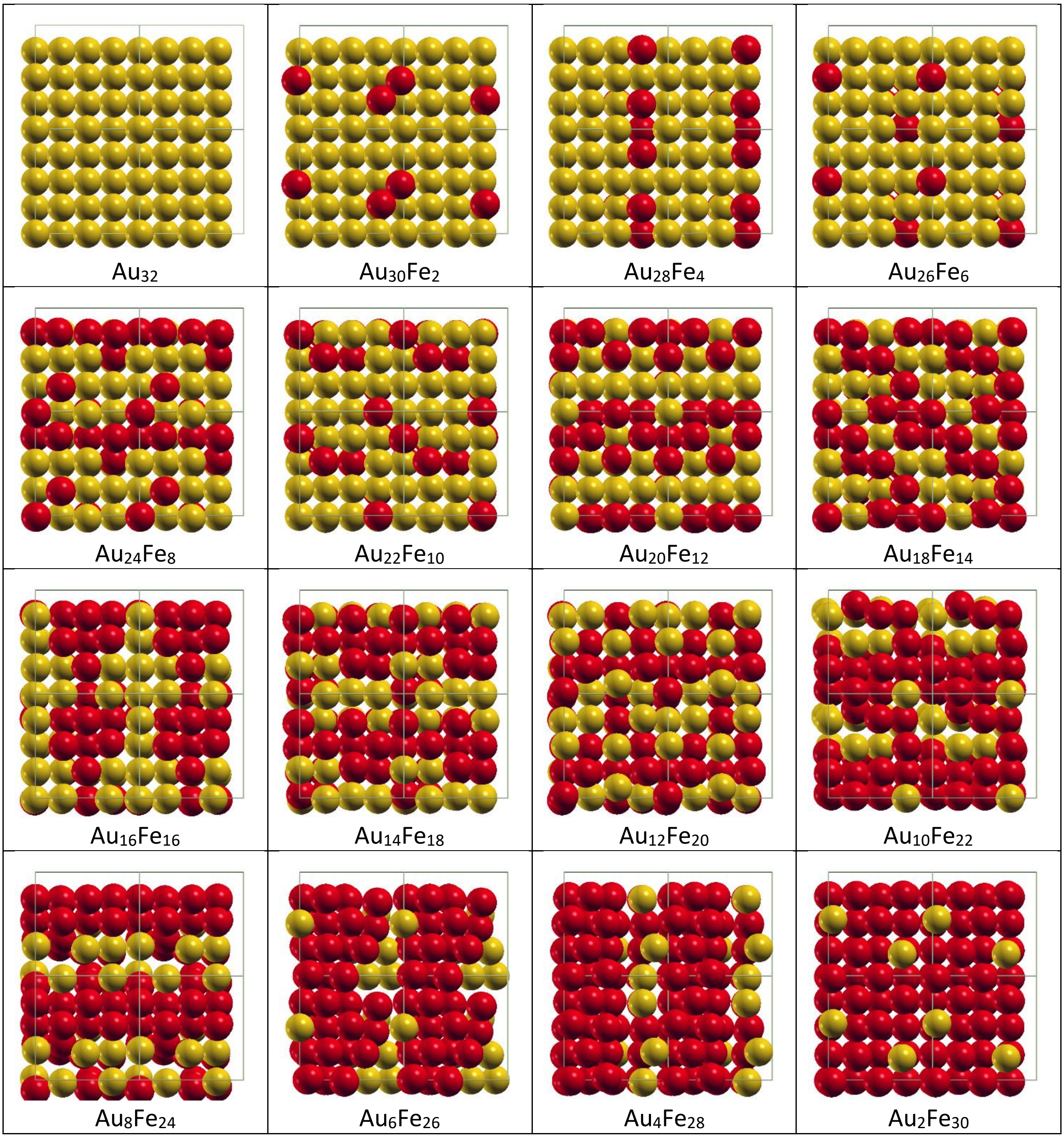

To better understand trends in the mixing enthalpy and crystal structure of the alloy, we ran two series of calculations: (i) geometry optimizations where the cell parameter is kept frozen at the proper value up to a concentration of atomic Fe of 20%, while for larger concentration the cell parameter is fixed at the one obtained for $Au_{80\%}Fe_{20\%}$, and (ii) variable cell optimizations of the alloy models. Results are shown in table S5.

Formation energies of the alloy were computed as

$$E_{form} = E_{tot} - (n_{Au} \times E_{Au,bulk} + n_{Fe} \times E_{Fe,bulk})$$

As expected, the mixture of Fe and Au is energetically disfavored. Moreover, formation energies increase monotonically with the amount of Fe even for very large amount of Fe, because the cell parameter is fixed to the value of $Au_{80\%}Fe_{20\%}$, and Fe is constrained in an unphysical fcc lattice. When the cell parameter is free to relax (*i.e.* the cell is allowed to shrink also for Fe concentration beyond 20%), the formation energy shows a maximum at ~60% Fe, and our computed values compare well with formation enthalpies of the Au-Fe solid solution obtained from thermodynamic data[16] (see Figure S3 and Figure 1 in main text).

Table S5: Outcomes of total energy calculations of SQS alloy models. Both Variable-Cell Relaxation (VCR) and Fixed-Cell Relaxation (FCR) results are reported. Formation energies are calculated as described in text. The last column shows the cell c/a ratio, which maps the transition from a fcc to a bcc lattice.

Fe%	Formation energy / eV FCR	cell parameter / Å FCR	Formation energy / eV VCR	cell parameter / Å VCR	c/a VCR
0.00%	0.00	8.313	0.00	8.313	(fcc) 1.000
6.25%	1.17	8.270	1.17	8.286	0.993
12.50%	1.63	8.228	1.61	8.233	0.993
18.75%	2.17	8.186	2.13	8.167	0.999
25.00%	2.94	8.178	2.76	8.146	0.986
31.25%	3.77	8.178	3.24	8.100	0.983
37.50%	4.67	8.178	4.65	7.997	0.992
43.75%	5.64	8.178	4.01	7.961	0.986
50.00%	6.45	8.178	4.48	7.821	0.993
56.25%	8.36	8.178	4.65	7.821	0.986
62.50%	9.66	8.178	4.52	7.885	0.942
68.75%	11.06	8.178	4.27	8.157	0.822
75.00%	12.71	8.178	3.87	8.266	0.764
81.25%	14.60	8.178	2.93	8.283	0.734
87.50%	16.68	8.178	2.05	8.231	0.724
93.75%	19.61	8.178	2.37	7.916	0.764
100.00%	0.00	8.015	0.00	8.015	(bcc) 0.707

Figure S3: mixing enthalpy per atom as function of the Fe atomic percentage. Orange squares: calculated, blue circles: from thermodynamic data [16], green triangles: parameterized (see Section "Topological model of dealloying")

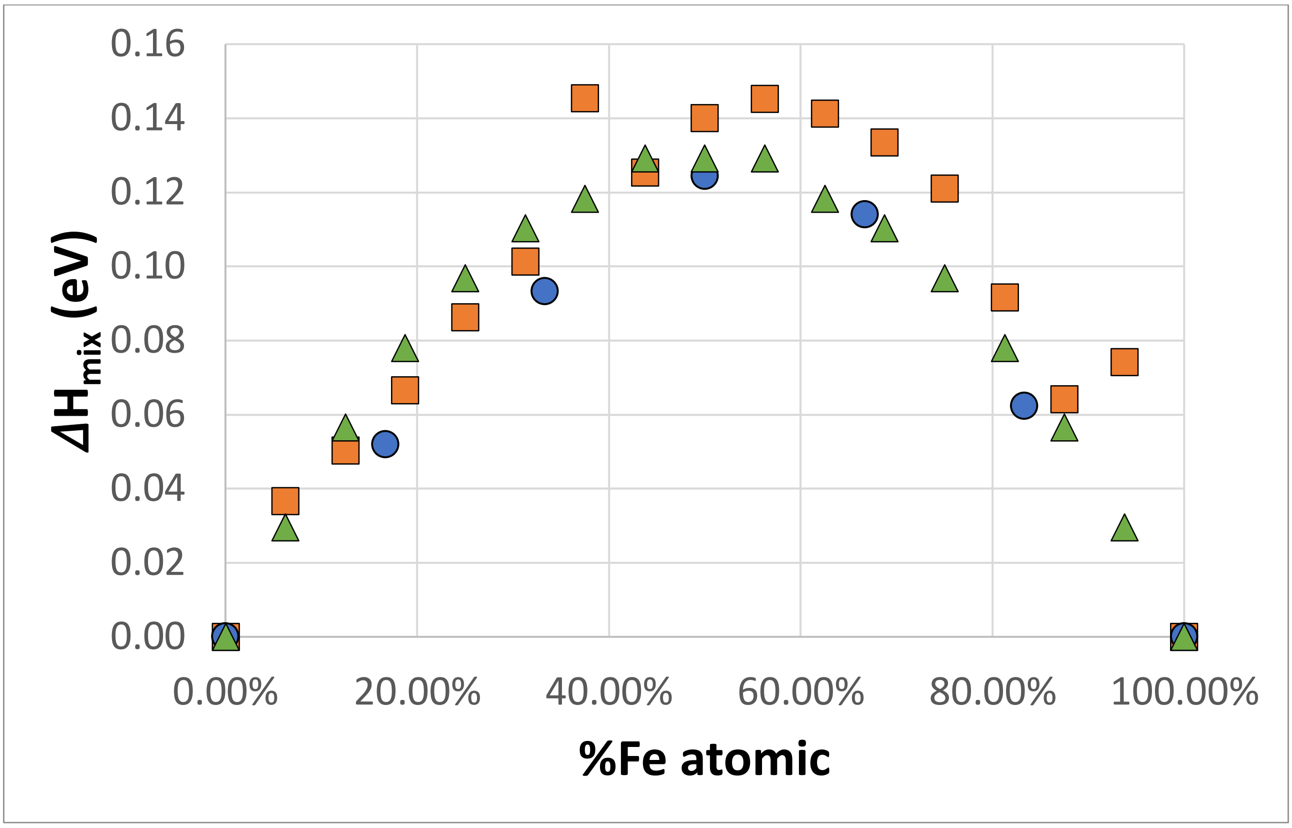

S5. Topological model of dealloying:

The observation that Fe embedded in an Au matrix can be oxidized effectively under experimental conditions raises the question of whether or not the oxidation can propagate beneath the surface and lead to extended corrosion.

From an atomistic point of view, corrosion can happen through different mechanisms: (i) propagation of the oxidation by means of oxygen migration through the alloy lattice, (ii) Fe migration towards the surface to sustain superficial oxidation, or (iii) pitting of the surface and consequent exposure of surface Fe to the aqueous environment.

The first two mechanisms are frozen at room temperature owing to the large energy barrier towards diffusion (table S4). For pitting to happen, the presence of Fe percolation paths is required, whose appearance in the alloy clearly depends on the amount of Fe. In fact, binary alloys can undergo extended dealloying of the less noble metal only if the amount of such a component is large enough to avoid surface passivation by the more noble metal. Artymowicz *et al.* [17] developed a topological model to identify the parting limit for dealloying of an AuAg alloy. Their idea was that dealloying requires the presence of percolation paths that span through the bulk material. To identify whether these structures are present for a certain alloy composition, they generated ensembles of random alloy slab models and performed a topological analysis to identify percolation paths that span the slab from side to side. Such paths are composed by Ag atoms, *i.e.* the less noble component in the alloy, surrounded by *m* or more Ag sites. The best results were obtained for *m* equal to 8 or 9. The percentage of models that presents at least one percolation path ramps sharply from 0% to 100% around the percolation threshold, which makes it easy to identify.

Since Au and Ag are completely miscible at any ratio, the topological analysis of Ref.[17] was performed on ensembles of purely random alloy models. In contrast, Au and Fe form non-equilibrium alloys and tend to segregate. We take the tendency of forming elementally-pure clusters into account by considering the mixing enthalpy for the formation of the alloy. We use a simple parametric model of mixing enthalpy where each Fe nearest-neighbour to an Au atom contributes an energy of 0.043 eV, a value based on DFT calculations using SQS models (Fig. S3). The probability of substitution of an Au with Fe is then given by a Boltzmann factor $e^{-\frac{\Delta H_{mix}}{\tau}}$, where τ is a parameter that measures the "thermal" disorder of the alloy. With this choice, the substitution of Au with Fe is always accepted if energetically favoured, that is when $\Delta H_{mix} \leq 0$. If the mixing enthalpy is positive, the substitution is accepted or not accordingly to the computed probability. The τ parameter controls the degree of mixing of Au and Fe in the alloy: for $\tau \rightarrow \infty$ the alloy tends to a solid solution, while by lowering the τ parameter the mixing of Au and Fe is disfavoured and at the same time the presence of nearest neighbors of the same kind is promoted. In other words, the Boltzmann factor can be seen as a term controlling the population of the alloy states: the Au-Fe mixing has a higher enthalpy than the sum of pure Au and pure Fe, then it requires energy to be populated. By increasing the value of τ the mixing of Au and Fe is progressively promoted.

Slab model were generated multiplying the fcc primitive cell by the same integer along each direction and then applying periodic boundary conditions in any two directions. We performed the topological analysis on slabs with atomic thickness 64 (~15nm), 96 (~22nm), or 128 (~30nm) by varying the amount of Fe in the alloy. In agreement with previous calculations[17] the percolation threshold of the solid solution is 53% (59%) of the less noble component for *m*=8 (*m*=9). The amount of Fe needed to produce percolation paths drops to lower values when the presence of Fe-Fe nearest-neighbors (Figure 1E, reported below in an extended version as Figure S4).

Figure S4: percentage of alloy models with percolation paths as a function of the atomic percentage of Fe and the thermal disorder parameter τ. Percolation path is defined for the case m=8 (see text).

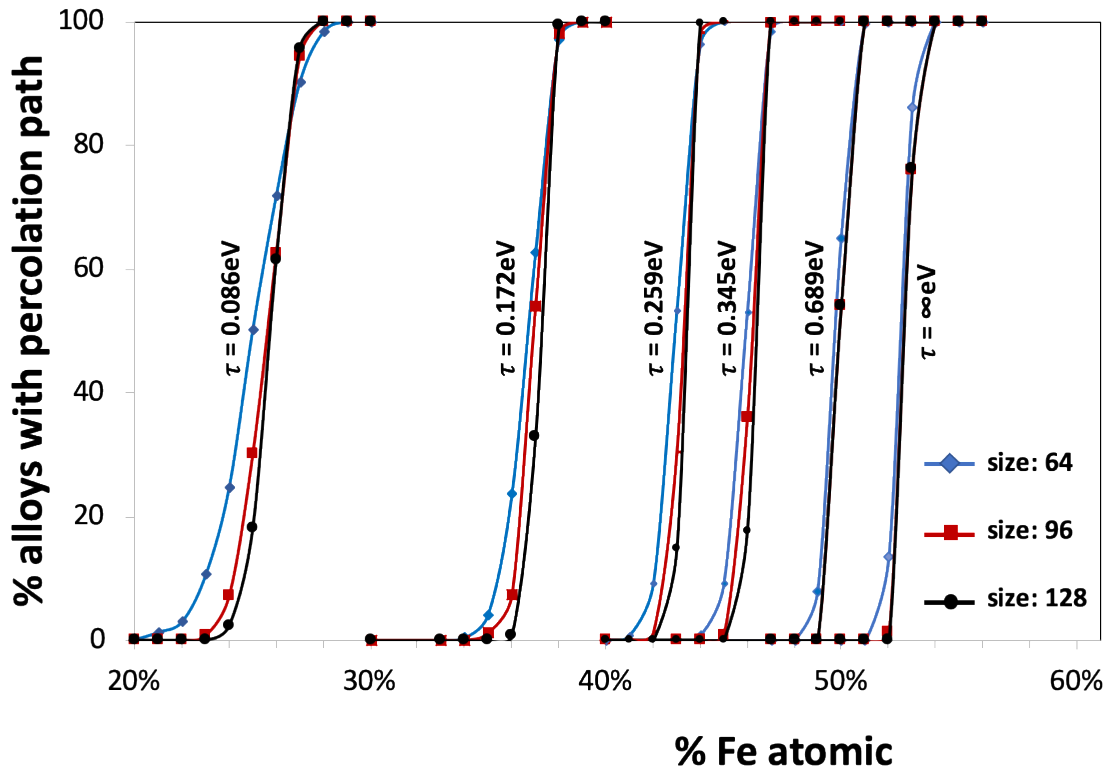

Table S6: Computed percolation threshold vs τ (size: 64)

τ (eV)	Threshold (Fe at%)
0.08617	25
0.17235	37
0.25852	43
0.34469	46
0.68939	50
∞	53

Experimental data

S6. Additional structural characterization data

Table S7. Composition of the Au-Fe nanoparticles as evaluated by ICP-MS on a set of ≥ 3 independent batches.

Sample	Au at% ± s.d.	Fe at% ± s.d.
**Au(79)Fe(21)**	79 ± 1	21 ± 1
**Au(70)Fe(30)**	70 ± 4	30 ± 4
**Au(50)Fe(50)**	50 ± 6	50 ± 6

Table S8. Lattice parameters estimated by Le Bail refinement of the XRD patterns and average crystallite size obtained by the Scherrer formula. Where a component due to a disordered phase is present, the cell parameter is referred only to the crystalline components. Where more than one crystalline component is present, the cell parameters are reported in order of relative intensity.

Sample	Lattice parameter *a* (Å)	Average crystallite size (nm)
**After the synthetic procedure**		
**Au**	4.079	14
**Au(79)Fe(21)**	4.005	18
**Au(70)Fe(30)**	3.986	16
**Au(50)Fe(50)**	3.984	9
**Post 2 months in PBS (pH 7.4)**		
**Au**	4.078	11
**Au(79)Fe(21)**	4.005 – 4.042	20 - 6
**Au(70)Fe(30)**	4.075 - 3.985	7 - 8
**Au(50)Fe(50)**	4.076 - 3.986	7 - 6
**Post 2 months in citrate buffer (pH 4.7)**		
**Au**	4.079	8
**Au(79)Fe(21)**	4.008 - 4.038	22 - 6
**Au(70)Fe(30)**	3.994 - 4.064	8 - 4
**Au(50)Fe(50)**	4.078	4

Figure S5. XRD spectra (30 – 130 2θ range) of the four samples as obtained after the synthetic procedure (A), and after 2 months incubation in PBS (B) or citrate buffer (C) at 37 °C. The fits according to the Le Bail model are reported in green. Peaks denoted with * are due to buffer salts and included in the refinement fitting to improve the accuracy wherever their intensity was comparable to the main reflections of the metal phase.

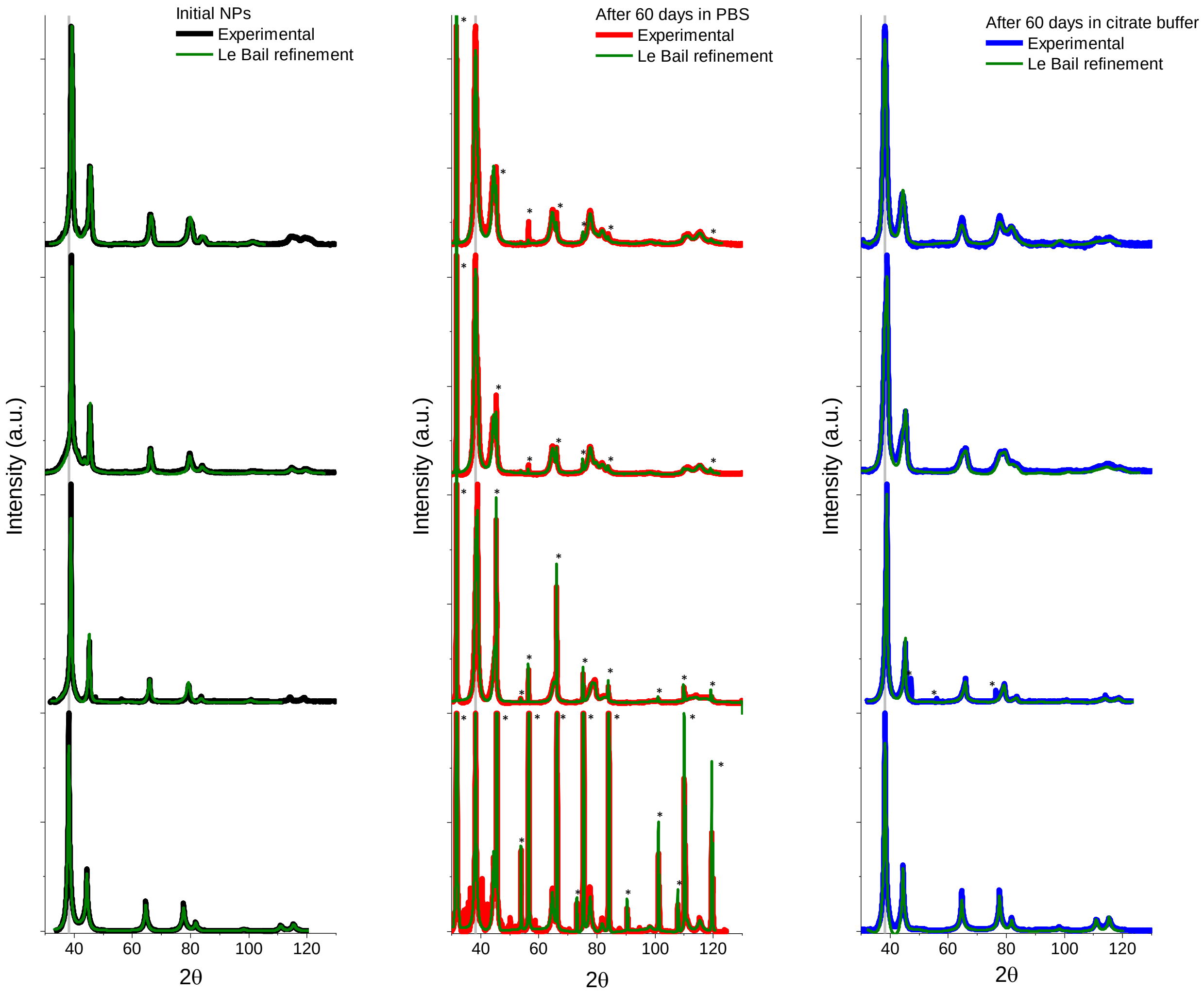

Figure S6. HRTEM images of Au(50)Fe(50) NPs, showing a polycrystalline structure with high defect density. Inhomogeneous electronic contrast is appreciable in the NPs, suggesting the presence of non-homogeneous element distribution, as expected from enthalpy-weighted alloy models.

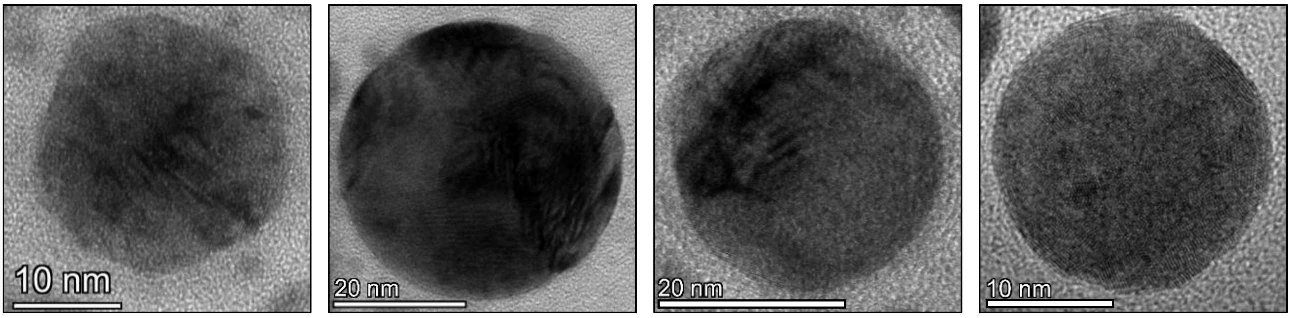

Figure S7. STEM-HAADF-DF images (top) and STEM-EDS maps of Au M-line (middle) and Fe K-line (bottom) of Au(50)Fe(50) NPs. The DF images agrees with the HRTEM analysis of Figure S6, showing the presence of a non-homogeneous contrast (*i.e.* a non-homogeneous distribution of elements with different atomic number) inside the NPs. Nonetheless, the bidimensional element maps confirm the coexistence of Au and Fe inside the alloy NPs, that was already pointed out by the XRD analysis.

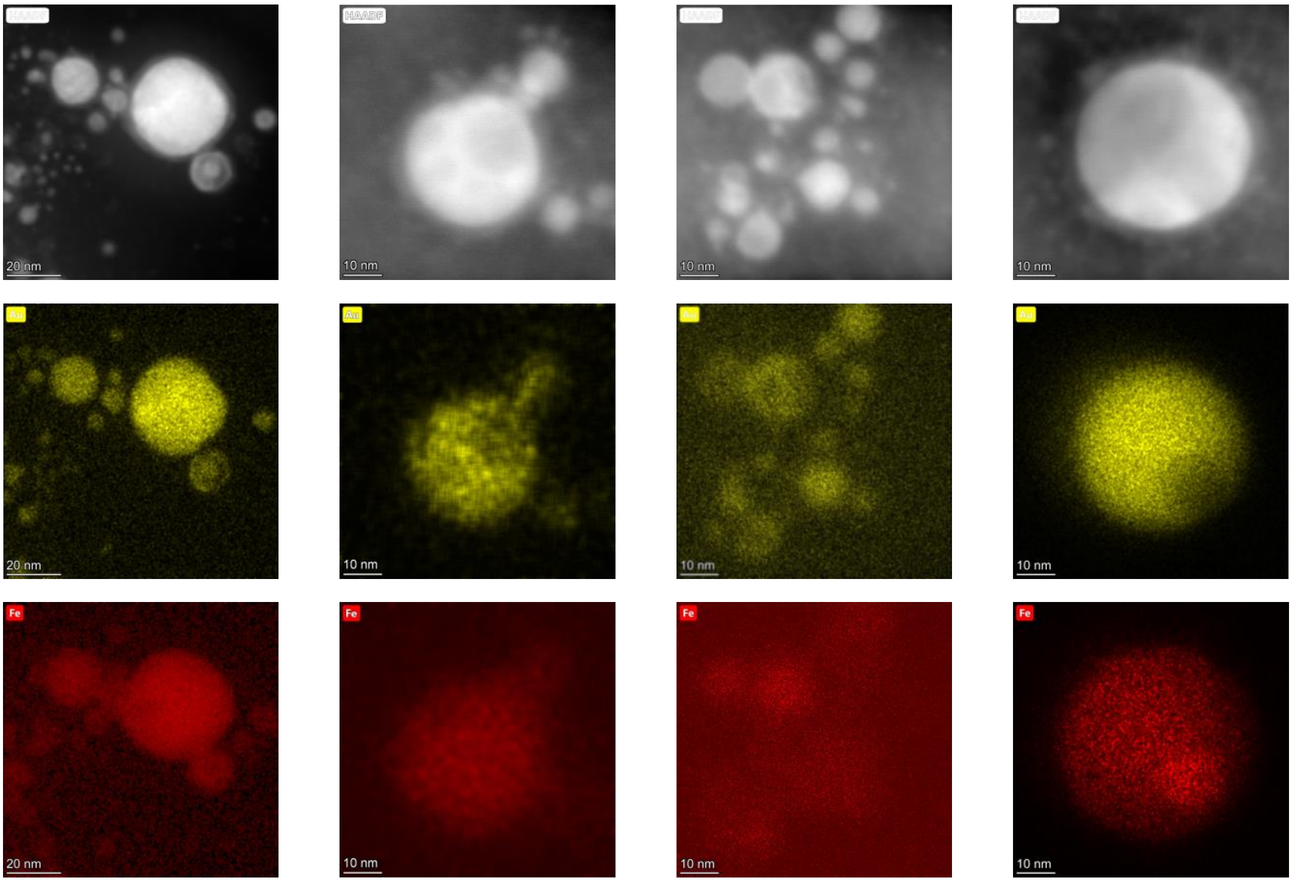

Figure S8. Size distributions obtained by fitting of the SAXS curves collected before (black lines) and after (red lines) incubation of the four samples in distilled water at 37 °C for 2 months (1440 h).

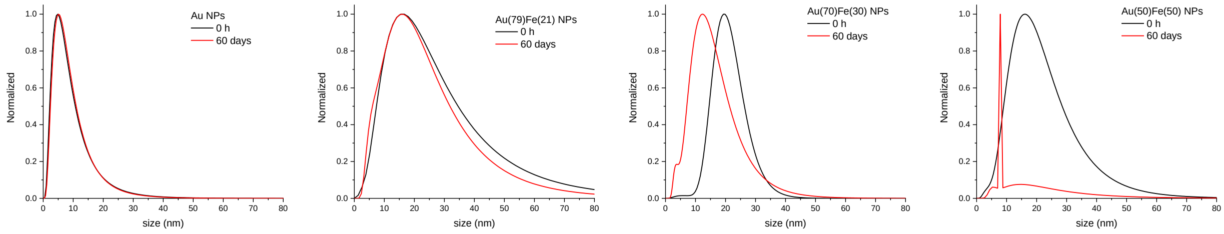

Figure S9. Size histograms of the Au(50)Fe(50) NPs incubated in FCS at ph 7.4 or 4.7. The plot of the average size is reported in Fig. 3A.

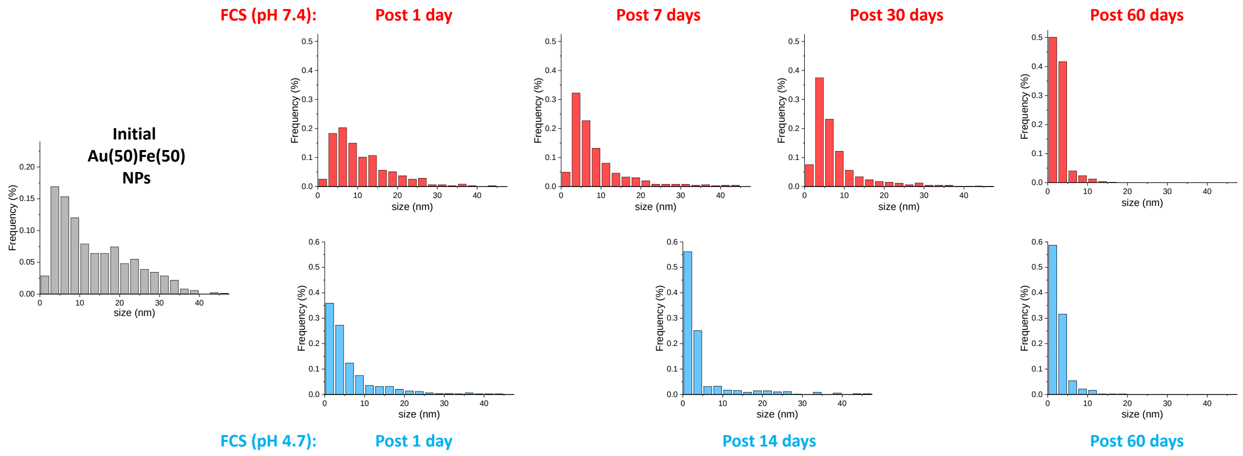

Figure S10. Quantification of Fe and Au in liquid solution of Au(50)Fe(50) NPs incubated at 37 °C in FCS at pH 7.4 (red squares) or pH 4.7 (blue circles) for different time-points. After each timepoint, the solution was dialysed with a 3 kDa concentration membrane and analysed with ICP-MS. The results confirm that degradation of NPs is faster at lysosomal pH than in physiological conditions, explaining the faster increase of dissolved Fe in the samples. Instead, a negligible fraction of Au is found, meaning that this metal is not released as free ions or any other chemical substance smaller than the dialysis threshold of 3 kDa.

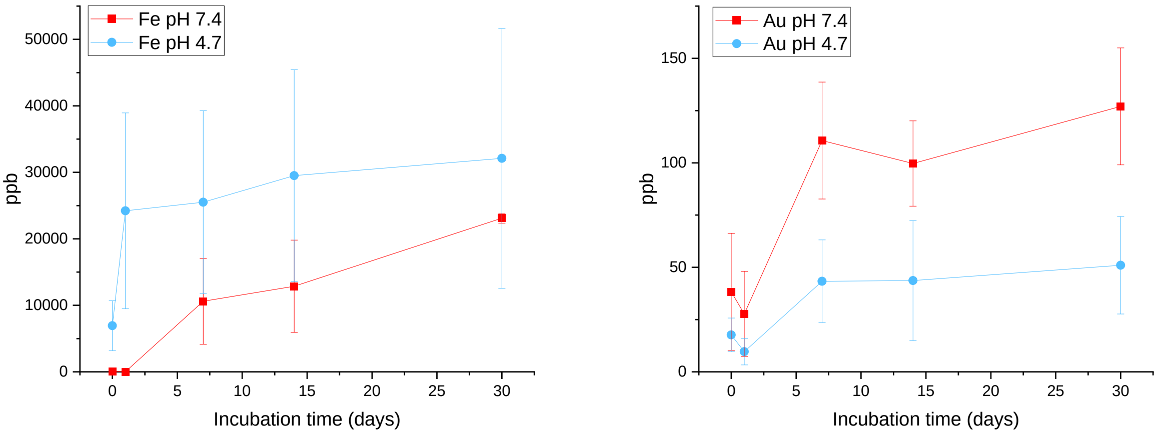

Figure S11. Hydrodynamic diameter of Au(50)Fe(50) NPs after 1 h (black line) and 24 h (red line) in FCS.

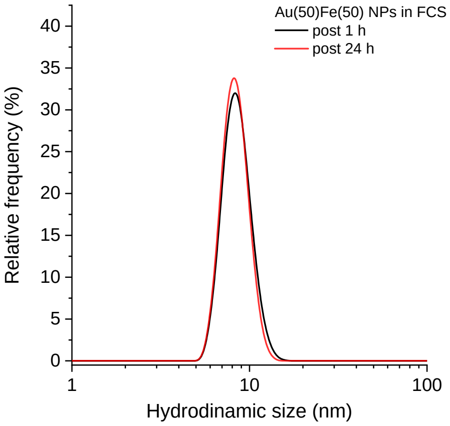

Figure S12. Powder FTIR spectra of physical mixtures of PEG and BSA with various proportions. All spectra were normalized to the PEG C–O–C stretching at 1100 cm^{-1} and shifted for clarity.

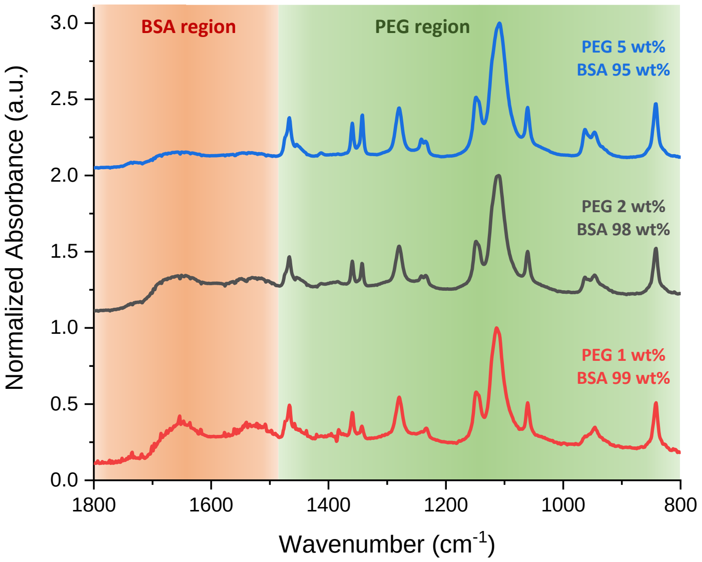

S7. Additional *in vivo* biodistribution data

Figure S13. CT images of bladder region in mice before and 78 days after administration of Au(50)Fe(50) NPs or Au NPs.

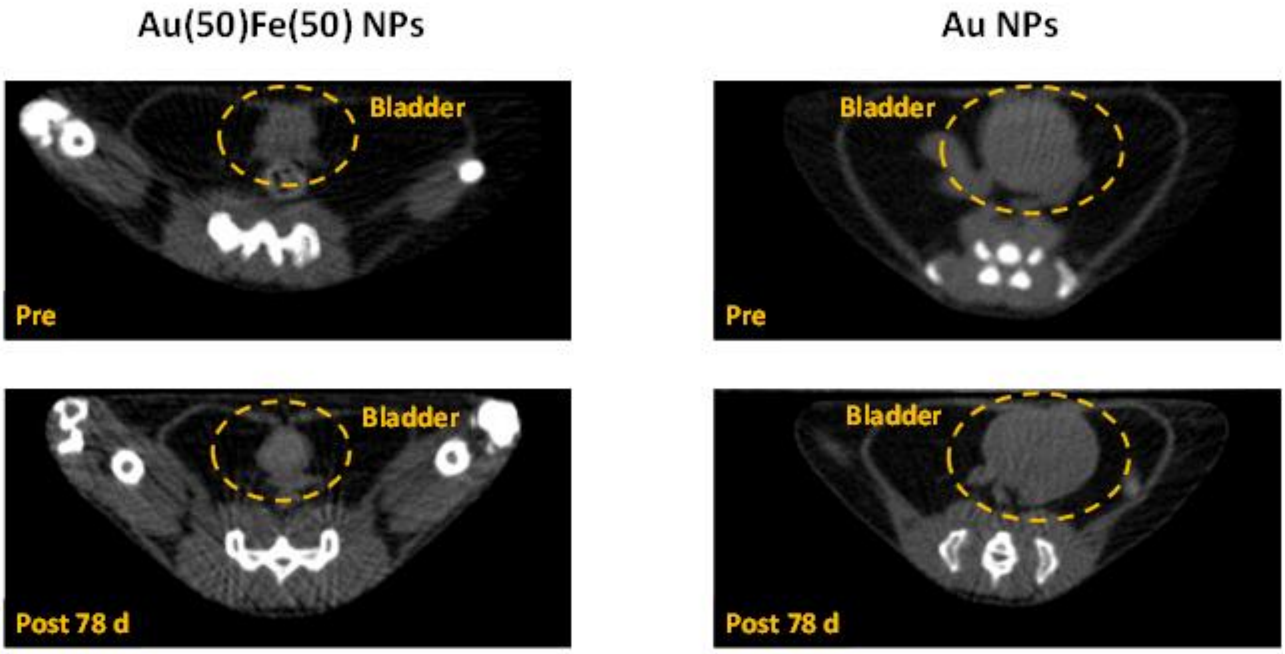

Figure S14. T_2-weighted MRI images of bladder region in mice before and 30 days after administration of Au(50)Fe(50) NPs or Endorem.

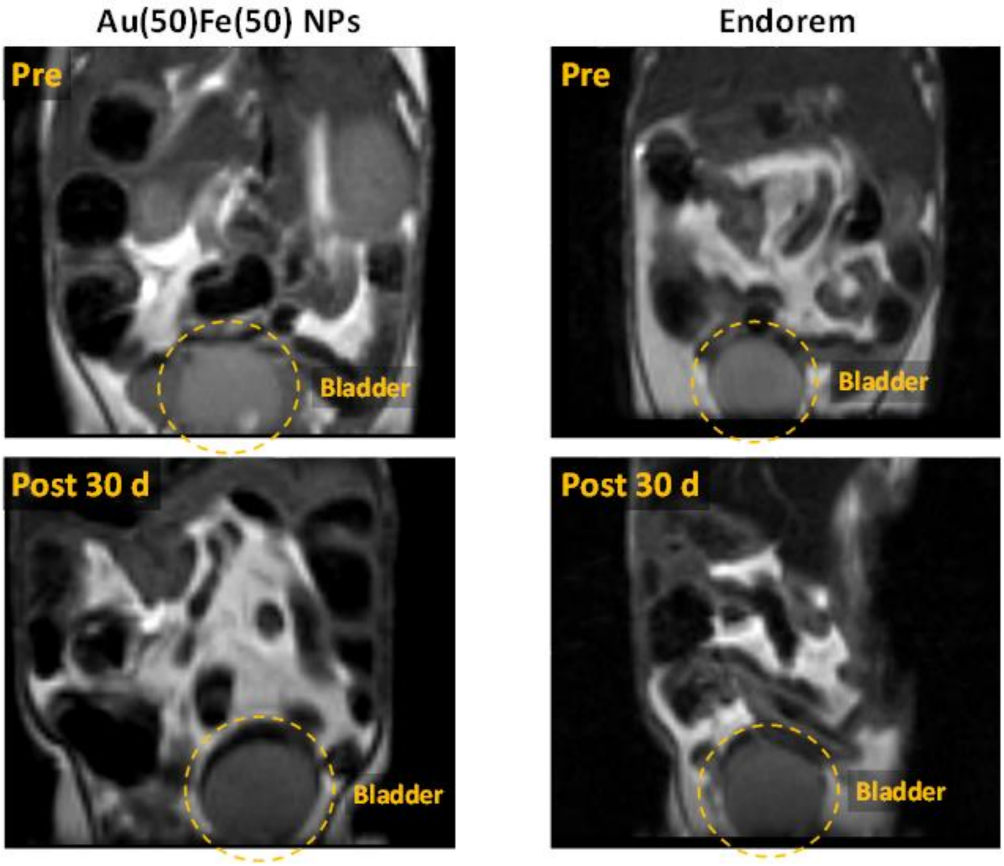

Figure S15. Histopathological analysis on liver, spleen and kidneys of mice treated with Au(50)Fe(50) and Au NPs for 78 days (*in vivo* CT experiment). The upper panel refers to the tissues treated with the iron-stain kit, where accumulation of ionic iron is detectable as blue spots. The lower panel refers to the tissues treated with the hematoxylin-eosin (HE) stain kit. Dark spots are due to clusters of metal nanoparticles and are especially evident in spleen, partly evident in liver and rare or not detected in kidneys. Spleen and liver of the Au NPs treated mice show higher density of nanoparticles clusters compared to the Au(50)Fe(50) NPs treated mice.

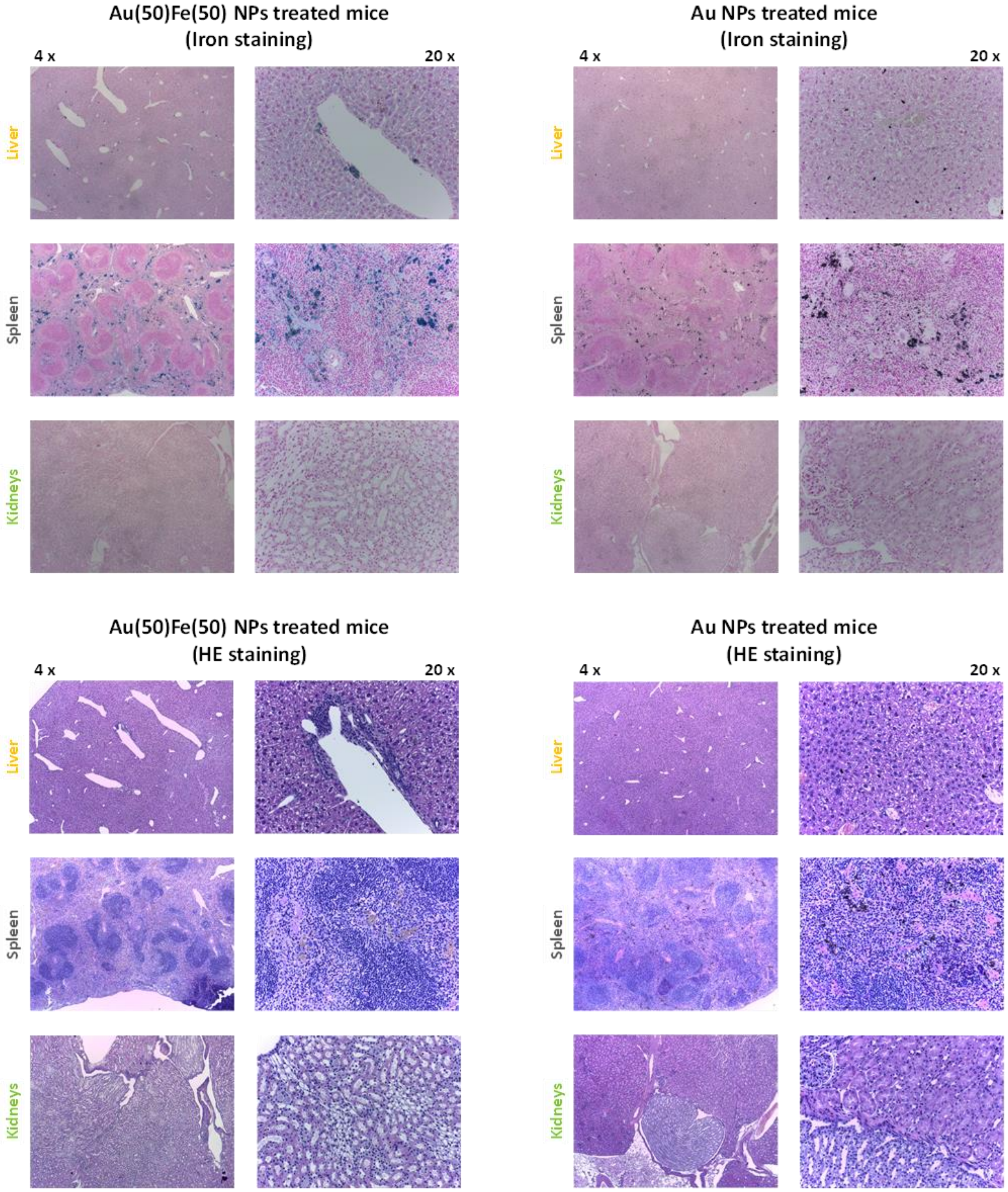

Figure S16. ESEM evaluation of liver, spleen and kidney tissues (same of the histopathological analysis, iron staining treated) in the Au(50)Fe(50) NPs and Au NPs treated mice.

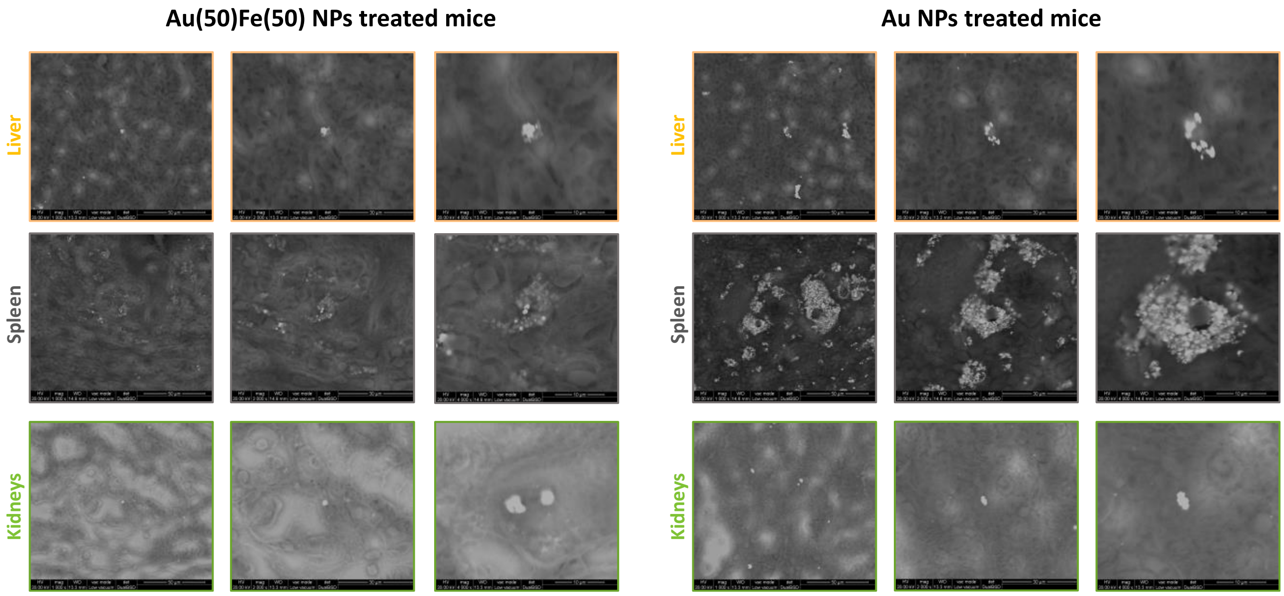

Bibliography

(1) Giannozzi, P.; Andreussi, O.; Brumme, T.; Bunau, O.; Buongiorno Nardelli, M.; Calandra, M.; Car, R.; Cavazzoni, C.; Ceresoli, D.; Cococcioni, M.; Colonna, N.; Carnimeo, I.; Dal Corso, A.; de Gironcoli, S.; Delugas, P.; Di Stasio, R. A.; Ferretti, A.; Floris, A.; Fratesi, G.; Fugallo, G.; *et al.* Advanced Capabilities for Materials Modelling with Quantum ESPRESSO. *J. Phys.: Condens. Matter* **2017**, *29*, 465901.

(2) Perdew, J. P.; Burke, K.; Ernzerhof, M. Generalized Gradient Approximation Made Simple. *Phys. Rev. Lett.* **1996**, *77*, 3865–3868..

(3) Garrity, K. F.; Bennett, J. W.; Rabe, K. M.; Vanderbilt, D. Pseudopotentials for High-Throughput DFT Calculations. *Comput. Mater. Sci.* **2014**, *81*, 446–452.

(4) Marx, D.; Hutter, J. *Ab Initio* Molecular Dynamics: Basic Theory and Advanced Methods; Cambridge University Press, Cambridge, **2009**

(5) Björneholm, O.; Hansen, M. H.; Hodgson, A.; Liu, L.-M.; Limmer, D. T.; Michaelides, A.; Pedevilla, P.; Rossmeisl, J.; Shen, H.; Tocci, G.; Tyrode, E.; Walz, M.-M.; Werner, J.; Bluhm, H. Water at Interfaces. *Chem. Rev.* **2016**, *116*, 7698–7726.

(6) Wang, L.-L.; Johnson, D. D. Predicted Trends of Core-Shell Preferences for 132 Late Transition-Metal Binary-Alloy Nanoparticles. *J. Am. Chem. Soc.* **2009**, *131*, 14023.

(7) Tran, R.; Xu, Z.; Radhakrishnan, B.; Winston, D.; Sun, W.; Persson, K. A.; Ong, S. P. Surface Energies of Elemental Crystals. *Sci. Data* **2016**, *3*, 160080.

(8) Nørskov, J. K.; Rossmeisl, J.; Logadottir, A.; Lindqvist, L.; Kitchin, J. R.; Bligaard, T.; Jónsson, H. Origin of the Overpotential for Oxygen Reduction at a Fuel-Cell Cathode. *J. Phys. Chem. B* **2004**, *108*, 17886–17892.

(9) Anderson, A. B.; Uddin, J.; Jinnouchi, R. Solvation and Zero-Point-Energy Effects on OH(Ads) Reduction on Pt(111) Electrodes. *J. Phys. Chem. C* **2010**, *114*, 14946–14952.

(10) Linstrom, P. J.; Mallard, W. G. *NIST Chemistry Webbook*; National Institute of Standards and Technology: Gaithersburg, 2018.

(11) Henkelman, G.; Jónsson, H. Improved Tangent Estimate in the Nudged Elastic Band Method for Finding Minimum Energy Paths and Saddle Points. *J. Chem. Phys.* **2000**, *113*, 9978–9985.

(12) Whittle, G. L.; Campbell, S. J. Local Atomic Order in AuFe. *Phys. Rev. B* **1984**, *30* (9), 5364.

(13) Duhl, D.; Hirano, K.-I.; Cohen, M. Diffusion of Iron, Cobalt and Nickel in Gold. *Acta Metall.* **1963**, *11*, 1–6.

(14) Mantina, M.; Wang, Y.; Chen, L. Q.; Liu, Z. K.; Wolverton, C. First Principles Impurity Diffusion Coefficients. *Acta Mater.* **2009**, *57*, 4102–4108.

(15) Von Pezold, J.; Dick, A.; Friák, M.; Neugebauer, J. Generation and Performance of Special Quasirandom Structures for Studying the Elastic Properties of Random Alloys: Application to Al-Ti. *Phys. Rev. B* **2010**, *81*, 094203.

(16) Boom, R.; De Boer, F. R.; Niessen, A. K.; Miedema, A. R. Enthalpies of Formation of Liquid and Solid Binary Alloys Based on 3d Metals: III. Alloys of Iron. *Phys. B* **1983**, *115*, 285–309.

(17) Artymowicz, D. M.; Erlebacher, J.; Newman, R. C. Relationship between the Parting Limit for De-Alloying and a Particular Geometric High-Density Site Percolation Threshold. *Philos. Mag.* **2009**, *89*, 1663–1693.
